# Hce2 domain‐containing effectors contribute to the full virulence of* Valsa mali* in a redundant manner

**DOI:** 10.1111/mpp.12796

**Published:** 2019-03-26

**Authors:** Mian Zhang, Shichang Xie, Yuhuan Zhao, Xiang Meng, Linlin Song, Hao Feng, Lili Huang

**Affiliations:** ^1^ State Key Laboratory of Crop Stress Biology for Arid Areas and College of Plant Protection Northwest A&F University Yangling China

**Keywords:** Apple *Valsa* canker, effector proteins, Hce2 domain, tandem copy, virulence factor

## Abstract

*Valsa mali* is the causal agent of apple *Valsa *canker, a destructive disease in East Asia. Effector proteins play important roles in the virulence of phytopathogenic fungi, and we identified five Hce2 domain‐containing effectors (VmHEP1, VmHEP2, VmHEP3, VmHEP4 and VmHEP5) from the *V.* *mali* genome. Amongst these, VmHEP1 and VmHEP2 were found to be up‐regulated during the early infection stage and VmHEP1 was also identified as a cell death inducer through its transient expression in *Nicotiana benthamiana*. Although the deletion of each single *VmHEP* gene did not lead to a reduction in virulence, the double‐deletion of *VmHEP1* and *VmHEP2* notably attenuated *V. mali* virulence in both apple twigs and leaves. An evolutionary analysis revealed that *VmHEP1 and VmHEP2* are two paralogues, under purifying selection. *VmHEP1* and *VmHEP2* are located next to each other on chromosome 11 as tandem genes with only a 604 bp physical distance. Interestingly, the deletion of *VmHEP1* promoted the expression of *VmHEP2* and, vice versa, the deletion of *VmHEP2* promoted the expression of *VmHEP1*. The present results provide insights into the functions of Hce2 domain‐containing effectors acting as virulence factors of *V. mali*, and provide a new perspective regarding the contribution of tandem genes to the virulence of phytopathogenic fungi.

## Introduction

For survival, plants have evolved a sophisticated defence system that recognizes invading pathogens and induces a cascade of distinct events to resist invaders (Zipfel, 2014). Pathogens also have strategies to answer these challenges and suppress plant defences by secreting diverse effector proteins into their host. The recognition of these effector proteins by the plants result in a renewal of hostile responses, and effectors then escape plant recognition (Albert 2013; Boller and He, 2009). Those extremely skilful responses and countermeasures used by plants and pathogens are classically regarded as a well‐bedded interactive systems known as PAMP‐triggered immunity (PTI), effector‐triggered susceptibility (ETS) and effector‐triggered immunity (ETI) (Dodds and Rathjen, 2010; Jones and Dangl, 2006). Although this classic model has recently been challenged by the invasion model (Cook *et al*., 2015), effector proteins are still considered to play important roles in host‐pathogen interactions.

For successful infection, effectors secreted by pathogens manipulate the host plants and subvert their immune responses. A uniform definition for effectors remains elusive, and more microbial‐related molecules with typical effector characteristics, such as small RNAs and other small secreted molecules, have been identified (Lo Presti *et al*., 2015; Rovenich *et al*., 2014; Wang *et al*., 2016). In a narrow definition, some secreted proteins are termed effector proteins, which are generally defined as small secreted proteins containing at most 300 amino acids rich in cysteine residues, which are very important for the structure of disulfide bridges (Duplessis *et al*., 2011; Muller *et al*., 2008; Stergiopoulos *et al*., 2012). Some effector proteins share conserved functional domains, but the diversity of effector proteins leads to varied modes of action. Some effector proteins act as interferential factors of the oxidation‐reduction system. For example, Pep1 from* Ustilago maydis*, which attacks the maize peroxidase POX12 to suppress the early oxidative burst process (Hemetsberger *et al*., 2012); peroxidase‐like effectors VmPODs from *V. mali* contribute to full pathogen virulence (Feng et al., 2018). Some target plant proteases, such as NIP1 and NIP3 from *Rhynchosporium commune*, target the plasma membrane H^+^‐ATPase (Hahn *et al*., 1993; Wevelsiep *et al*., 1991, 1993). Moreover, pathogen effectors, such as the effector PsAvr3c from *Phytophthora* *sojae*, can reprogramme host pre‐mRNA splicing to promote infection (Huang *et al*., 2017). In addition to directly attacking important host targets, a previous study found that the effector PsXLP1 from *P. sojae *works as a disguiser to shield the true virulence factor (Ma *et al*., 2017). The important roles of effectors draw sustained attention, and many studies are continuously identifying novel effectors and revealing their mechanisms. However, due to their substantial diversity and quantity, most of their mechanisms remain unknown, and many potential effectors urgently need to be identified (Lo Presti *et al*., 2015; Wang *et al*., 2014).

Most of the fungal effectors revealed thus far are species specific, and the Hce2 domain is one of the few conserved domains for effectors and has been defined as a domain that causes plant cell death. Hce2, an ancient and widely conserved domain distributed in the fungal kingdom, plays a central role in some effector proteins that promote virulence functions in a diverse set of hosts, and its name was derived from homologues of *Cladosporium fulvum* Ecp2 effector protein (Stergiopoulos *et al*., 2012). The Ecp2 effector protein is reported as a 165 amino acid protein secreted to the host apoplast that was originally found in the tomato pathogen *C.* *fulvum* and was observed to contribute to its virulence (Laugé *et al*., 1997). Homologues of Ecp2 were later found in the banana pathogen *Mycosphaerella fijiensis *and the wheat pathogen *Mycosphaerella graminicola* (Stergiopoulos *et al*., 2010), and further exploration of 135 fungal genomes shows that Ecp2 exists widely in fungi (Stergiopoulos *et al*., 2012). In general, the diversity and widespread distribution of Hce2 domain‐containing effectors in different species suggest that the Hce2 domain is a crucial factor for survival in differentiated ecological habitats (Stergiopoulos *et al*., 2012).

*V.* *mali *is a necrotrophic fungus that mainly infects apple trunks with conidia via wounds and ultimately causes the destructive disease apple *Valsa* canker in East Asia. Large economic losses are incurred each year in China, and strategies for complete disease prevention and control remain limited (Ke *et al*., 2013; Wang *et al*., 2011). In a previous study, several candidate proteins that could suppress BAX‐induced cell death were identified through transient expression in *Nicotiana benthamiana* (Li *et al*., 2015), and two virulence‐contributing effectors, VmEP1 and VmPxE1, were recently identified (Li *et al*., 2015; Zhang *et al*., 2018). However, an analysis of genome information databases revealed 779 proteins secreted by *V. mali*, and many of these show potential effector features (Li *et al*., 2015; Yin *et al*., 2015). Thus, the available information regarding *V. mali* effectors remains very limited.

In this research, Hce2 domain‐containing effectors of *V. mali* and their contribution were determined, which provides a new understanding and perspective for Hce2 domain.

## Results

### Five Hce2 domain‐containing effector proteins were identified from the genome of *V.* *mali*

**Figure 1:**
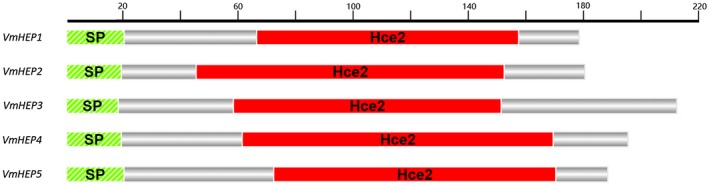
Schematic diagrams of five Hce2 domain‐containing effectors identified from *V. mali*. The green colour shows the position and segment of the predicted signal peptides, and the red colour indicates the Hce2 domain.

The Hce2 domain was identified from homologues of the *C. fulvum* Ecp2 effector protein, which are widely distributed in the fungi kingdom, and is defined as a domain that can cause plant cell death. Through an analysis of the *V. mali* genome, we identified five genes with significant and unique hits to the Hce2 hidden Markov model (HMM) (PF14856, model length of 102 amino acid). The alignment length of these hits ranged from 86 to 101 amino acid (Table S1). A total of 99 full‐length proteins (453 sequences from 169 fungal species), which constructed the model, were homologous to *V. mali* Hce2s (Table S2). Within *V. mali*, the five Hce2s were homologous to each other and showed a protein sequence identity between 22% and 36% (Table S2). We found that two Hce2 homologues, VmHEP1 and VmHEP2, which showed the higher similarity compared with the other homologues, were encoded by tandem genes in the same orientation, 604 bp apart. These five secreted *V. mali* proteins with significant and unique hits (Table S1) had an HMM alignment length between 86 amino acid and 101 amino acid. VmHEP1 (KUI73808.1) contained 179 amino acids and an Hce2 domain from 67^th^–158^th ^amino acid; VmHEP2 (KUI73807.1) contained 181 amino acids and an Hce2 domain from 46^th^–153^rd ^amino acid; VmHEP3 (KUI69607.1) consisted of 213 amino acids and contained an Hce2 domain from 59^th^–152^nd ^amino acid; VmHEP4 (KUI71688.1) contained 196 amino acids and an Hce2 domain from 62^nd^–170^th ^amino acid; and VmHEP5 (KUI65020.1) comprised 189 amino acids and an Hce2 domain from 73^rd^–171^st^ amino acid. Each of the five effectors contained a predicted signal peptide and an Hce2 domain (Fig. 1).

### *VmHEP1 *and *VmHEP2 *are symparalogs (in‐paralogous) genes under significant purifying selection

**Figure 2:**
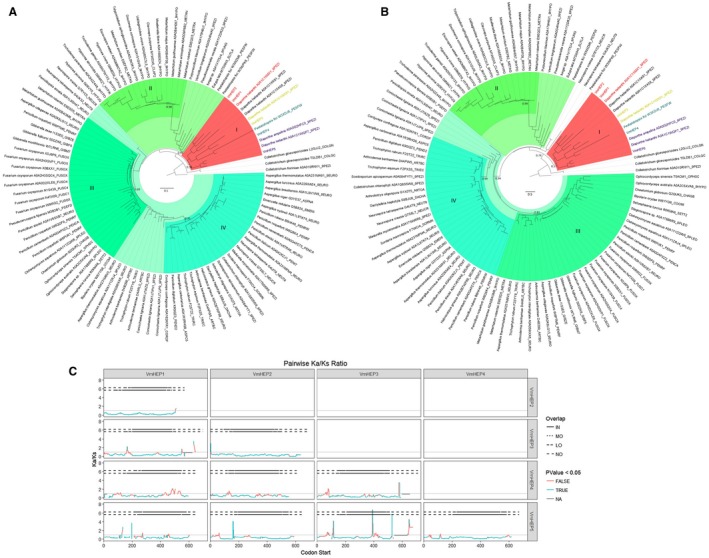
Phylogenetic analysis and selective pressure analysis of Hce2 domain‐containing effectors. (A) The phylogenetic trees of full‐length sequences and (B) Hce2 domains. The most related branches to VmHEPs are shown with different colours, and the tip names are the UniProt accession ID of each Hce2 protein. (C) Depiction of the Ka/Ks ratio distribution of each pairwise comparison. For each box in the figure, the x‐axis represents the site of the start codon of a 60‐base length window in alignment; the y‐axis shows the calculated Ka/Ks ratio of the window, colours of the lines designate a significant difference (fisher *P*‐value < 0.05) between the ratio and 1, NA indicates that the *P*‐value could not be estimated; and the horizontal dotted line indicates overlap of the window and the Hce2 domain region. IN: inside the domain; MO: overlap size more than 30 bases; LO: less than 30 bases; NO: outside the domain. (i.e. the solid part of the dotted line indicates that the window was completely inside the Hce2 domain region).

Both the amino acid sequence phylogenetic analysis and the domain phylogenetic analysis revealed that the Hce2 domain‐containing effectors in *V. mali *were not well clustered with each other, with the exception of *VmHEP1* and *VmHEP2. *This result indicated that *VmHEP1* and *VmHEP2 *were symparalogs (in‐paralogous), whereas the other three genes were alloparalogous (out‐paralogous) (Fig. 2A,B). Additionally, a pairwise selective pressure analysis demonstrated significant purifying selection between *VmHEP1* and *VmHEP2* (Ka/Ks < 1, *P* < 0.05). In contrast, the other pairwise comparisons revealed that (*VmHEP3*, *VmHEP4* and *VmHEP5*) were under positive selection (Ka/Ks > 1, *P* < 0.05), and this positive selection occurred outside the Hce2 domain (Fig. 2C). Furthermore, both full‐length and domain‐level phylogenetic analyses could almost divide the Hce2 genes into four subclades (Fig. 2A,B). All Hce2s from *V. mali* belonged to subclade I, and it is likely that duplication events occurred before or after the separation of *V. mali* from its ancestor, because these genes are highly similar to Hce2 from *Diaporthe helianthi*, which causes stem canker in sunflowers, and all Hce2s with the exception of *VmHEP1 *and *VmHEP2* were scattered in subclade I.

### Relative transcription level of VmHEP effector genes

**Figure 3:**
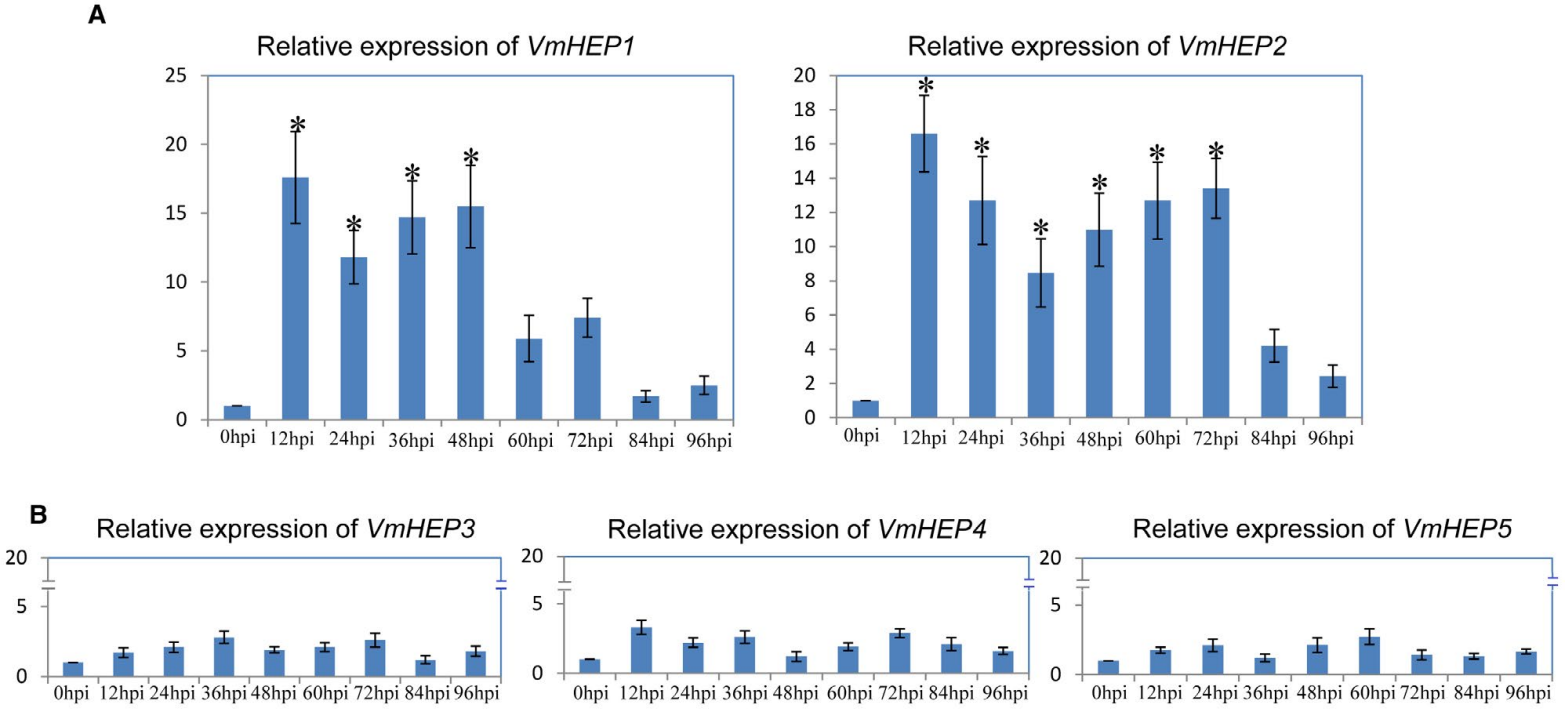
Relative expression level of *VmHEPs* at different time points and conditions post‐inoculation of apple twigs, and *G6PDH *of *V. mali* was used as the housekeeping gene. (A) Relative expression levels of *VmHEP1* and *VmHEP2.*, (B) *VmHEP3,* *VmHEP4 *and *VmHEP5 *post‐inoculation of apple twigs at 0, 12, 24, 36, 48, 60, 72, 84 and 96 hpi. Each *VmHEP* gene was measured relative to the transcription level of the corresponding *VmHEP* genes at 0 hpi (the transcription level of each *VmHEP* gene at 0 hpi was set to 1). The results are presented as the mean fold changes in expression relative to the expression at 0 h. Significant differences are indicated with asterisks (*P* < 0.05). Each experiment was repeated twice, and error bars indicate standard errors of the means (SEMs).

Quantitative Reverse Transcription‐Polymerase Chain Reaction (qRT‐PCR) analysis at eight different time points (12, 24, 36, 48, 60, 72, 84 and 96 hpi) showed that during early infection, only the transcription levels of V*mHEP1 *and *VmHEP2 *were significantly up‐regulated (fold change > 5) relative to their respective expression 0 hpi (0 hpi corresponds to mycelium sampled immediately before inoculation). *VmHEP1* was noticeably up‐regulated at 12, 24, 36 and 48 hpi, and *VmHEP2* was significantly up‐regulated at 12, 24, 36, 48, 60 and 72 hpi (Fig. 3A). Notably, both *VmHEP1 *and *VmHEP2 *were particularly up‐regulated at 12 hpi (fold change > 15). During the early infection stage, the up‐regulation trend of *VmHEP3*, *VmHEP4* and *VmHEP5* was not detected relative to each of their expression levels at 0 hpi (0 hpi corresponds to mycelium sampled immediately before inoculation) (Fig. 3B).

### Validation of the secretory function of the putative N‐terminal signal peptide of *VmHEPs*

**Figure 4:**
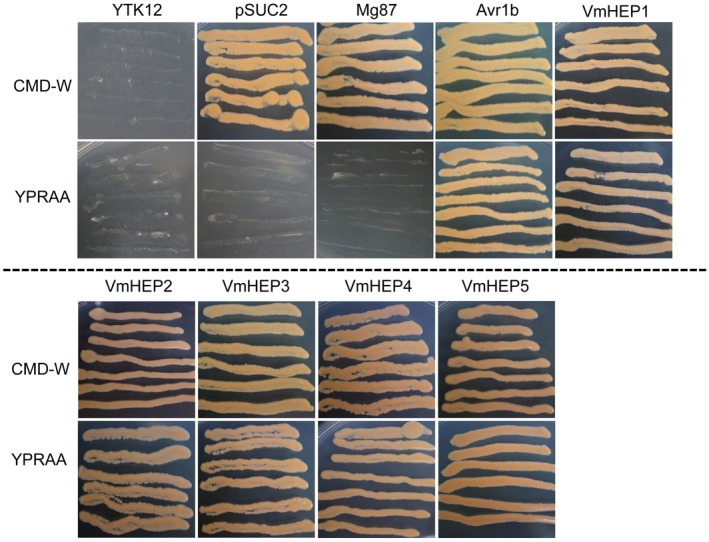
Secretory function validation of the putative N‐terminal signal peptides of VmHEPs using the yeast invertase secretion assay. (A) Schematic diagrams of putative signal peptides of *VmHEPs*. (B) Only yeast strain YTK12 transformed with the pSUC2 plasmid containing the functional signal peptide secreted invertase and could grow on both CMD‐W and YPRAA media. The secreted effector Avr1b from oomycetes and the *Magnaporthe oryzae* non‐secreted Mg87 protein were regarded as positive and negative controls, respectively.

Each of the *VmHEPs* was predicted to contain a signal peptide: *VmHEP1*, 1^st^–21^st^ amino acid; *VmHEP2*, 1^st^–23^rd^ amino acid; *VmHEP3*, 1^st^–19^th^ amino acid; *VmHEP4*, 1^st^–20^th^ amino acid; and *VmHEP5*, 1^st^–21^st^ amino acid (Fig. 1). The function of these predicted signal peptides were verified through the yeast invertase secretion assay. The yeast invertase secretion assay was performed to verify the function of these predicted signal peptides. The invertase mutant yeast strain YTK12 containing the pSUC2 vector recombined with the signal peptides VmHEP1, VmHEP2, VmHEP3, VmHEP4 and VmHEP5 or the positive control Avr1b was able to grow on both CMD‐W and YPRAA media (with raffinose instead of sucrose), which indicated recovery of the deficiency in the secretory function of YTK12 and secretion of the invertase. These tested signal peptides were functional. In contrast, the negative control Mg87 showed obvious growth inhibition on YPRAA medium (Fig. 4).

### Transient expression of five candidate Hce2 domain‐containing effector proteins in *N. benthamiana*

**Figure 5:**
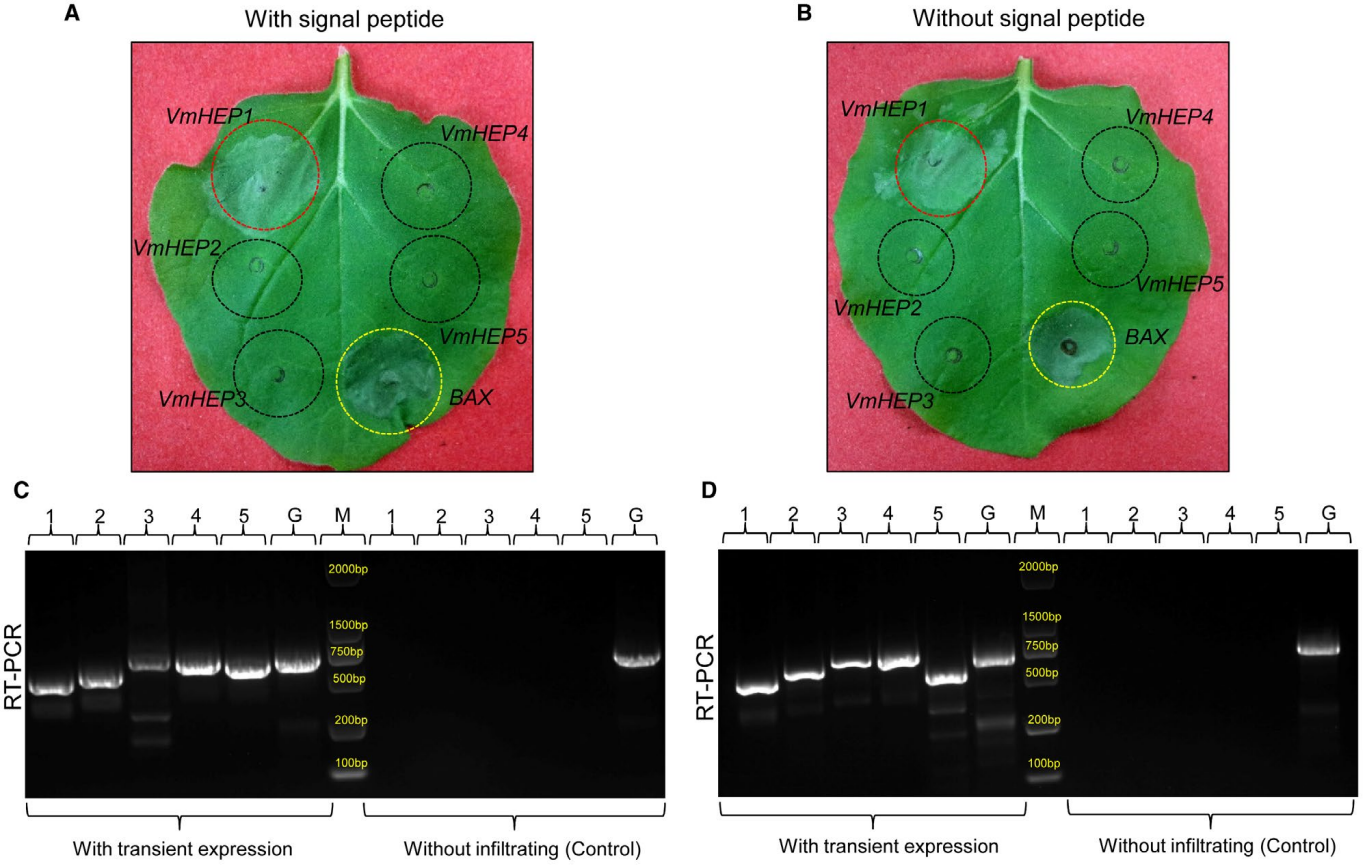
Symptoms after transient expression of five Hce2 domain‐containing effector *VmHEPs* (either with or without signal peptides) on the leaves of *N. benthamiana. *(A) Only VmHEP1 caused cell death in the leaves of *N. benthamiana. *(B) RT‐PCR successfully detected the expression of *VmHEPs* in *N. benthamiana *after the transient expression of *VmHEPs*, whereas Reverse Transcription‐Polymerase Chain Reaction (RT‐PCR) showed a negative result in *N. benthamiana *without transient expression. 1 : *VmHEP1*; 2 : *VmHEP2*; 3 :  *VmHEP3*; 4 :  *VmHEP4*; 5 :  *VmHEP5* and G :  *GAPDH *(*N. benthamiana*). This experiment was repeated twice. Each of the gene bands in the agarose gel shown by RT‐PCR was extracted and finally confirmed by gene sequencing.

The transient expression of five *VmHEPs *(either with or without signal peptide) in *N. benthamiana *showed that only *VmHEP1 *but not the other four *VmHEPs *could lead to cell death when transiently expressed in *N. benthamiana* cells 5 days after injection (Fig. 5A,B). The successful expression of each *VmHEP* gene in *N. benthamiana* was further confirmed by RT‐PCR (Fig. 5C,D). Each gene was tested on five *N. benthamiana *leaves, and the experiment was repeated twice. A total of 15 leaves were tested, and the symptoms were consistent. Each of the gene bands in an agarose gel shown by RT‐PCR was extracted and ultimately confirmed by gene sequencing.

### Deletion of individual *VmHEP *genes did not reduce the virulence of *V. mali*

**Figure 6:**
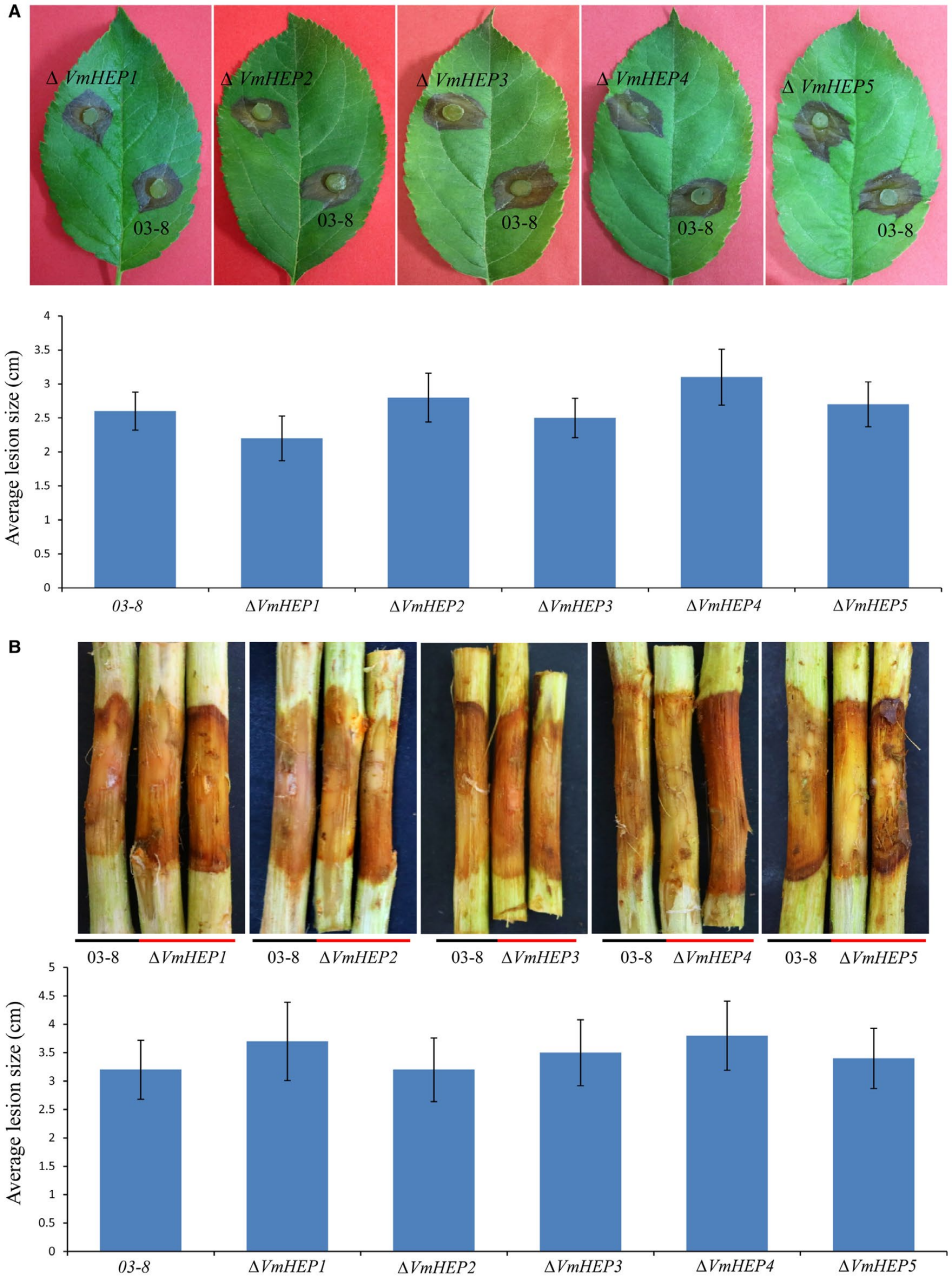
Symptoms on the leaves and twigs of *Malus domestica* Borkh. cv. Fuji after inoculation of *V. mali *wild‐type strain 03‐8 and each gene deletion mutant of *VmHEPs*. (A) No significant differences in virulence were detected on the leaves between *∆VmHEP1 *and 03‐8 (P_L1_ > 0.05), *∆VmHEP2 *and 03‐8 (P_L2_ > 0.05), *∆VmHEP3 *and 03‐8 (P_L3_ > 0.05), *∆VmHEP4 *and 03‐8 (P_L4_ > 0.05), and *∆VmHEP5 *and 03‐8 (P_L5_ > 0.05). (B) No significant differences in virulence were detected on the twigs between *∆VmHEP1 *and 03‐8 (P_T1_ > 0.05), *∆VmHEP2 *and 03‐8 (P_T1_ > 0.05), *∆VmHEP3 *and 03‐8 (P_T1_ > 0.05), *∆VmHEP4 *and 03‐8 (P_T1_ > 0.05), and *∆VmHEP5 *and 03‐8 (P_T1_ > 0.05). The evaluation of the leaves and twigs was performed 3 days and 5 days after inoculation, respectively. The bars indicate the standard deviations (SDs) of the mean of 30 individual host plants.

The contribution of each *VmHEP* to virulence was estimated through PEG‐mediated gene deletion. We successfully knocked out each of the five genes at an efficiency of 1/115, and it was confirmed by four pairs of PCR primers and Southern blotting (Fig. S1). The subsequent virulence test assays showed that no significant differences in virulence on leaves between *∆VmHEP1 *and 03‐8 (P_L1_ > 0.05), *∆VmHEP2 *and 03‐8 (P_L2_ > 0.05), *∆VmHEP3 *and 03‐8 (P_L3_ > 0.05), *∆VmHEP4 *and 03‐8 (P_L4_ > 0.05), and *∆VmHEP5 *and 03‐8 (P_L5_ > 0.05) (Fig. 6A). Furthermore, no significant differences in virulence on twigs were found between *∆VmHEP1 *and 03‐8 (P_T1_ > 0.05), *∆VmHEP2 *and 03‐8 (P_T1_ > 0.05), *∆VmHEP3 *and 03‐8 (P_T1_ > 0.05), *∆VmHEP4 *and 03‐8 (P_T1_ > 0.05), and *∆VmHEP5 *and 03‐8 (P_T1_ > 0.05) (Fig. 6B). These results indicated that a single deletion of the individual *VmHEP *genes did not influence virulence on either leaves or twigs.

### qRT‐PCR showed that VmHEP1 and VmHEP2 are complementary during infection

**Figure 7:**
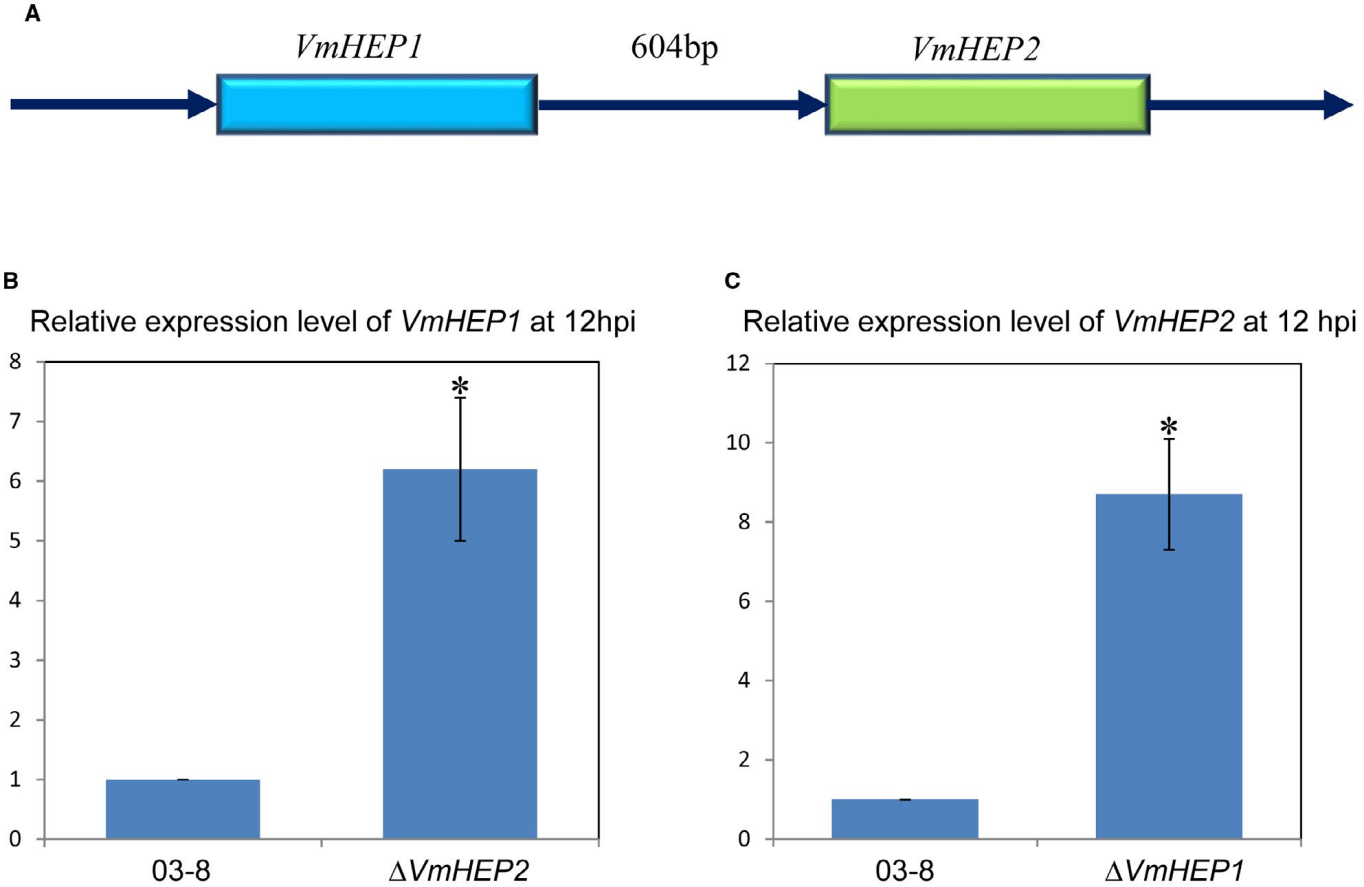
Relative expression levels of *VmHEP1* in* ∆VmHEP2 *and *VmHEP2* in *∆VmHEP1* compared to *VmHEP1 *or *VmHEP2 *in wild‐type 03‐8 at 12 hpi. (A) Schematic diagrams of the physical location between *VmHEP1 *and *VmHEP2 *on chromosome 11. (B) The relative expression level of *VmHEP1* in the deletion mutant *∆VmHEP2 *was significantly up‐regulated at 12 hpi (*P* < 0.05). (C) The relative expression level of *VmHEP2 *was also significantly increased (*P* < 0.05) in the deletion mutant* ∆VmHEP1 *at 12 hpi. The transcript levels of *VmHEP1/2* in wild‐type 03‐8 at 12 hpi were set to 1, and *G6PDH* (*V. mali*) was used as the housekeeping gene.

*VmHEP1* is located next to *VmHEP2* with only a 604 bp physical distance on chromosome 11. Furthermore, these two tandem genes are both located on the forward strand and have the same orientation (Fig. 7A). By means of qRT‐PCR analysis, at the non‐infection stage, the expression levels of *VmHEP1 *and* VmHEP2* showed no differences between the mutants *∆VmHEP2/1 *and wild‐type 03‐8 (Fig. S2). However, during infection, *VmHEP1* was significantly up‐regulated in the *VmHEP2*‐gene deletion mutant* ∆VmHEP2* relative to the expression level of *VmHEP1 *in wild‐type 03‐8. Moreover, *VmHEP2* was also significantly up‐regulated in the *VmHEP1*‐gene deletion mutant *∆VmHEP2* relative to the expression level of *VmHEP1 *in wild‐type 03‐8 (Fig. 7B). These data suggested that a deficiency in either one of these two tandem genes (*VmHEP1* and *VmHEP2*) could stimulate the expression of the other gene.

### *VmHEP1 *and *VmHEP2 *were not necessary growth factors of *V. mali*

Based on the abovementioned implications of the physical location and qRT‐PCR, we performed a double‐deletion of both *VmHEP1* and *VmHEP2*. Two double gene deletion mutants *∆VmHEP1*&*VmHEP2 *(#1 and #2) were generated successfully at an efficiency of 1/289 and confirmed by four pairs of PCR primers. Southern blot hybridization also showed that the imported resistance gene (*Hph* and *Neo*) was present as a single copy; which indicated that the changes in phenotype were not caused by multisite random insertion of the resistance gene (Figs S3 and 8C). We tested the growth of *∆VmHEP*&*VmHEP2 *on PDA (potato dextrose agar), PDA with 0.5 M KCl and PDA with 0.06 mol/L H_2_O_2_. The results showed that no significant difference in hyphal growth and stress resistance between the wild‐type 03‐8 and the double gene deletion mutant *∆VmHEP1*&*VmHEP2.* Further measurement of the conidiation ability also showed that there was no significant difference in the production of pycnidia between 03‐8 and *∆VmHEP1*&*VmHEP2 *(Supplementary Figs S4 and S5)*.*

**Figure 8:**
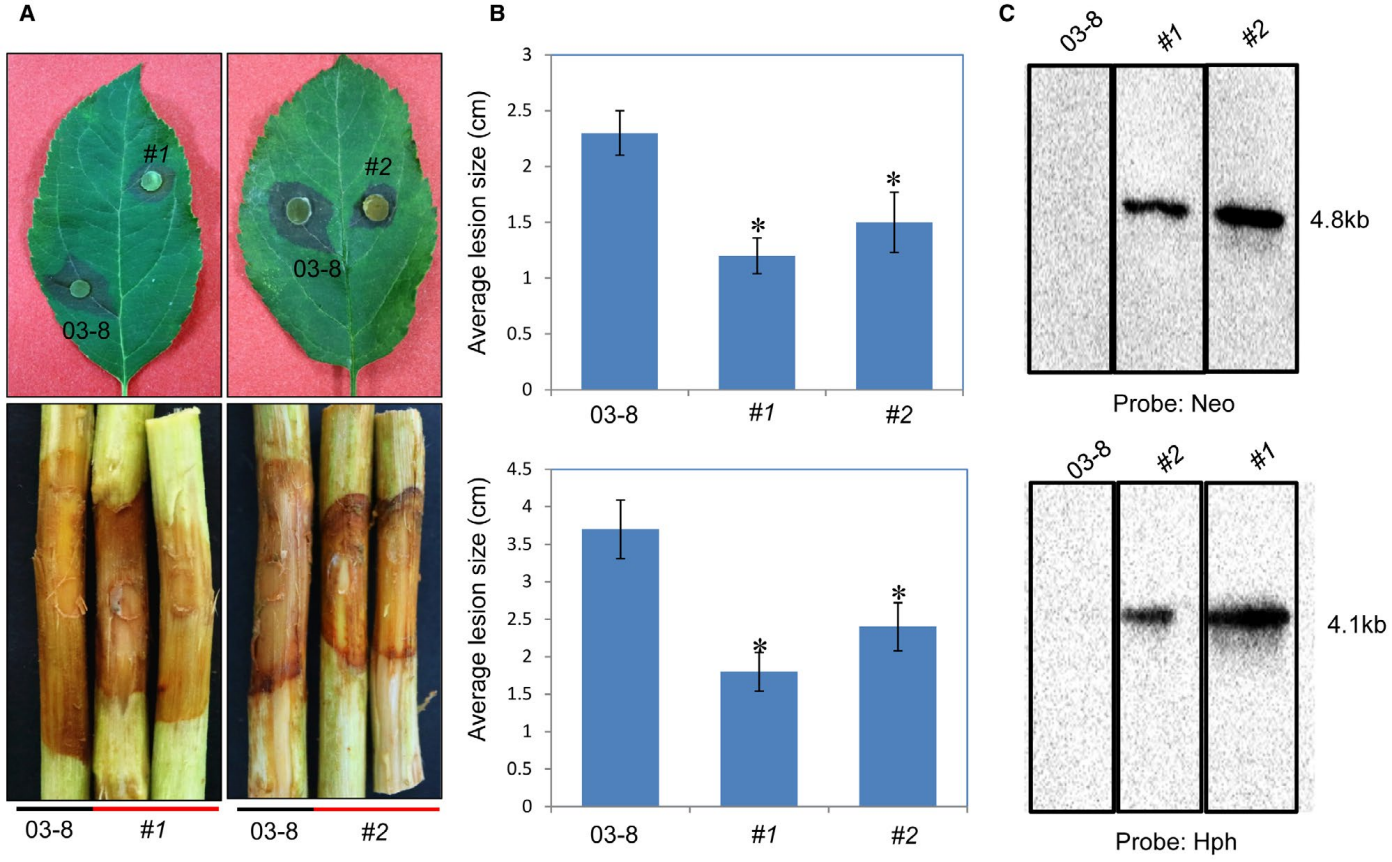
Pathogenicity assay of the *V. mali* wild‐type strain 03‐8 and double‐deletion mutant *∆VmHEP1&VmHEP2*, performed by inoculation onto leaves and twigs of *Malus domestica* Borkh. cv. Fuji. (A) Symptoms observed on twigs and leaves after inoculation. (B) Average lesion size of scabs measured on twigs and leaves. The evaluations on the leaves and twigs were performed 3 days and 5 days after inoculation, respectively. Asterisks indicate significant (*P* < 0.05) differences. The bars indicate the standard deviations (SDs) of the mean of 30 individual host plants. (C) Southern blot hybridization analysis of *∆VmHEP1*&*VmHEP2* mutants. The primers Neo‐F/Neo‐R and Hph‐F/Hph‐R with digoxin‐marked nucleic fragments of the *Neo* and *Hph* genes were used as the hybridization probes for gene deletion and copy number confirmation of the resistance genes, respectively.

### Double‐deletion of *VmHEP1 *and *VmHEP2 *significantly reduced the virulence of *V. mali*

Virulence was detected using the pathogenicity test. We inoculated the double‐deletion mutants on both detached apple (*Malus domestica* Borkh. cv. Fuji) twigs and leaves. Intriguingly, although single gene deletion did not lead to a reduction in virulence, a dramatic virulence reduction in the double‐deletion mutants (#1 and #2) was observed on both apple twigs and leaves (Fig. 8A), with a 30.4% average virulence decrease in leaves and a 27.8% average virulence decrease in twigs (Fig. 8B). The deficiency of *VmHEP1 *and *VmHEP2* led to a deficiency in *V. mali* infection, which indicated that these two genes act together as important virulence factors.

## Discussion

Effector proteins are important weapons used frequently to manipulate the immune responses of plants. Previous studies have provided strong evidence showing that effector proteins are abundant and crucial during the infection process of *V. mali* (Li *et al*., 2015). A previous study identified eight cell death suppressors (Li *et al*., 2015; Yin *et al*., 2015), whereas only one cell death inducer (VmHEP1) was identified in this study. As a necrotrophic fungus, *V. mali* is known to deploy cell death‐inducing effectors to destroy host cells as well as take over cell space and nutrients needed for survival. For example, *Botrytis cinerea *takes advantage of the SGT1‐mediated cell death immune signalling pathway to initiate its necrotrophic lifestyle (EI Oirdi and Bouarab, 2007). However, the amount of cell death suppressors is a new challenge for us to consider in addition to the traditional recognition of the behaviour of *V. mali*. We theorized that cell death‐inducing effectors are important for the acquisition of nutritional and ecological niches for *V. mali*, whereas cell death suppressors might compete with other necrotrophic competitors on apple trees. The suppression of host cell death could also suppress the colonization of other necrotrophic fungi. During fierce niche competition, the benefits of such actions might far outweigh the costs. Increasing lines of evidence shows that microorganisms deploy effectors to recruit cooperative microbes and thereby offer protection against microbial competitors for survival (Snelders *et al*., 2018). A previous qRT‐PCR analysis of eight cell death suppressors and three peroxidase‐like effectors from *V. mali* showed that the greatest up‐regulation of the expression of these effectors occurred at different time points (Feng *et al*., 2018; Li *et al*., 2015). Furthermore, in this study, we found that VmHEP1 (a cell death inducer) was significantly up‐regulated until 48 h post‐inoculation (hpi), which was longer than the up‐regulation of the eight cell death suppressors observed in the previous study. Thus, another explanation for these findings could be that these effectors play roles at different time points; specifically, it is possible that the effectors that show early up‐regulation suppress plant immunity and cell death, whereas those that are up‐regulated later in the process induce cell death.

The Hce2 domain was identified from homologues of *C. fulvum* Ecp2 effector protein and was previously reported to cause cell death. This domain was widely distributed in the fungi kingdom and has been identified as an important survival factor used by pathogenic fungi while dealing with the defensive system of their host and other competitors in a similar niche (Stergiopoulos *et al*., 2012). In this study, five Hce2 domain‐containing effectors (VmHEP1, VmHEP2, VmHEP3, VmHEP4 and VmHEP5) were identified in *V. mali*. An *in silico* analysis of these five Hce2 domain‐containing effectors showed that *VmHEP1* and *VmHEP2* were in‐paralogous, which implies that these two genes evolved from gene duplication in subsequent speciation events, whereas the other three were out‐paralogous, which indicated that the duplication preceded the speciation event. The in‐paralogous nature of *VmHEP1* and *VmHEP2* might indicate their importance in survival (Sonnhammer and Koonin, 2002), and the deletion of both of these gene in this study confirmed their necessity in the infection process of *V. mali*. Furthermore, purifying selection was detected between *VmHEP1* and *VmHEP2*, which indicates a conserved function (Fisher, 1999). However, the sequence identity between these two genes was only 36%, and only VmHEP1 could cause necrosis in the leaves of *N. benthamiana*. Based on the finding from a previous study that Ecp2 and its homologous effector protein MfEcp2 in *Mycosphaerella fijiensis, *could lead to different necrosis levels on different tomato lines containing different resistance genes (Stergiopoulos *et al*., 2010), we speculated that the sequence diversity of *VmHEP1* and *VmHEP2* would not affect the virulence function, but could help the pathogen avoid recognition by the host, and that the sequence variation was ensured successful infection even when confronted with different receptors from the host.

The single gene deletion of each *VmHEP* gene has no influence on virulence. Intriguingly, the double gene deletion of VmHEP1 and VmHEP2 showed a dramatic reduction in virulence, which implied a redundant function for these genes. This type of redundancy also exists in other effector families, such as the abundant RXLR‐containing effectors in oomycetes, which help oomycetes to reasonably distribute virulence and reduce the risk of failure of key effectors (Brich *et al*., 2008). Furthermore, a qRT‐PCR analysis of each gene showed a complementary effect between those two genes. If one of these two genes loses efficacy due to recognition or suppression by the host, *V. mali* could alter its invasion pattern to increase its recruitment of the other effector. This finding also suggests the existence of a potential communication mechanism between VmHEP1 and VmHEP2 during invasion that would improve performance and efficiency through mutual cooperation.

Furthermore, *VmHEP1* and *VmHEP2* are tandem duplicates located on chromosome 11 with an inter‐gene distance of only 604 bp. Some studies have emphasized the roles of tandem gene duplication in plants and animals (Cannon *et al*., 2004; Leister, 2004; Liu *et al*., 2008; Yanai *et al*., 1995). For example, a study on *Drosophila* indicates that tandem duplication causes overactivity of *alcohol dehydrogenase* (*Adh*) genes and might be an unidentified form of a position effect in the genome (Loehlin and Carroll, 2016). Thus, the tandem organization of *VmHEP1 *and *VmHEP2* guarantees gene function, which is consistent with the redundancy phenomenon observed in our study. Moreover, this result also implied that gene tandems constitute an important gene distribution pattern for the survival of phytopathogenic fungi. Gene tandems are likely generated by gene duplication, which is thought to play an important role in evolution and acts as an essential source of genetic novelty that ultimately leads to gene divergence. The divergence of the duplicated genes from their ancestor generally occurs through their neo‐functionalization or sub‐functionalization (He and Zhang, 2005; Lynch and Conery, 2000; Lynch and Force, 2000). For example, in fungi, these divergences contribute to the acquisition of special functions, such as the neo‐functionalization divergence of the effector Tin2 from *Ustilago maydis*, which is connected to the distinct pathogenic progress and lifestyle of this fungus (Tanaka *et al*., 2018), and the sub‐functionalization of the paralogous genes *BAT1* and *BAT2* in yeast, which regulates valine‐isoleucine‐leucine (VIL) biosynthesis (González *et al*., 2017). However, in *V. mali*, the general divergence process does not occur in Hce2‐containing proteins. In particular, *VmHEP1* and *VmHEP2*, the two most similar paralogues, are under purifying selection and play complementary roles during infection. The interrelation of *VmHEP1* and *VMHEP2* more likely corresponds to the pathogen's paralogous decoy partner, which facilitates effector invasion (Paulus and van der Hoorn, 2018). Similarly, PsXLP1, from *P. sojae*, acts as a decoy to shield the true virulence factor, which is a homologous gene with similar function (Ma *et al*., 2017). This strategy makes it more difficult for plants to protect themselves against invasion and activate disease resistance responses.

In this study, we revealed the function of five Hce2 domain‐containing effectors in *V. mali*, and our findings provide a new perspective for the contribution of tandem genes to virulence in phytopathogenic fungi.

## Experimental Procedures

### Strains and culture conditions

*N. benthamiana* plants were grown at 23 °C with a daily 14 h : 11 h light:  dark cycle in a culturing room. *Agrobacterium tumefaciens* strain GV3101 and *Escherichia coli* strain DH5α were cultured on Luria‐Bertani (LB) medium at 28 °C and 37 °C, respectively. Both *V. mali* strain 03‐8 and gene deletion mutants were cultured at 25 °C on PDA medium in an incubator. The material strains were stored in 15% glycerol at −70 °C in the Laboratory of Integrated Management of Plant Diseases at the College of Plant Protection, Northwest A&F University, Yangling, China.

### Plasmid constructs

Targeted genes were amplified from the cDNA library using Fast‐Pfu DNA polymerase (Takara, Dalian, China). PCR products (*VmHEP* genes or signal peptide sequences) were added to an A tail with Taq DNA Polymerase (Takara, Dalian, China) and cloned into a T‐simple vector by means of TA cloning (Takara, Dalian, China). Constructed plasmids were sequenced for confirmation by Sangon Biotech, Xi'an, China. The correct T‐*VmHEP* genes were digested with corresponding restriction enzymes (*Cla*I and *Sal*I), and the correct T‐signal peptide sequences were digested with corresponding restriction enzymes (*EcoR*I and *Xho*I) at 37 °C for 4 h. These VmHEP genes were finally cloned into the PGR106 (PVX) vector (*Cla*I and *Sal*I), and the signal peptide sequences were finally cloned into the pSUC2 vector (*EcoR*I and *Xho*I) with T4 DNA ligase at 22 °C for 3 h (Takara, Dalian, China) (Giraldo and Valent, 2013). The final constructed plasmids were sequenced for confirmation by Sangon Biotech, Xi'an, China.

### Sequence analyses and identification of Hce2‐containing effectors in *V. mali*

The whole‐genome shotgun sequences of *V. mali* were deposited at DDBJ/EMBL/GenBank under the accession JUIY01000000. SignalP 4.12 (http://www.cbs.dtu.dk/services/SignalP/) was used to predict the N‐terminal signal peptide (Yin *et al*., 2015). Pfam (http://pfam.xfam.org/) was used to predict the protein domain structure. To identify Hce2, the *V. mali* secretome was searched against the Pfam (v32.0) (Punta *et al*., 2000) database using hmmscan (hmmer3 version 3.1b2) implemented in pfam_scan.pl with the default parameters. The Hce2‐containing proteins in *V. mali* were identified using the HMM model of the Hce2 domain (PF14856, in the Pfam database) with the Perl script pfam_scan.pl. The proteins with significant hits (e‐value < 1e‐5) were considered Hce2 homologues.

### Phylogenetic analysis and selective pressure analysis of Hce2 domain‐containing effectors

All the full‐length sequences used to construct the HMM of the Hce2 domain were downloaded from the Pfam protein database (version 32.0) (Bateman *et al*., 2004), and VmHEPs were queried using BLAST (Altschul *et al*., 1997). The significant hit sequences (99 proteins, e‐value < 1e‐5) and the Hce2 domain region of these proteins were selected for phylogenetic analysis. Maximum likelihood (ML) methods, as implemented in RA x ML (v8.2.10) (Stamatakis, 2014), were used to infer the phylogenetic trees with 1000 bootstrap replicates. Before this, linsi from the software MAFFT (v7.402) (Katoh and Standley, 2013) performed the multiple sequence alignment, and then the WAG + I + G AA substitution matrix was selected by ProtTest (v 3.4.2) (Darriba *et al*., 2011). Finally, we used transfer bootstrap expectation (TBE) to estimate the support value of our phylogenetic trees (Lemoine *et al*., 2018). The Ka/Ks ratio was used to infer the direction and degree of natural selection acting on VmHEP genes. To do this, we selected the model selection method (‐m MS) as implemented in KaKs_Calculator (v2.0) (Wang *et al*., 2010), and a pairwise comparison of CDS sequences was completed by ParaAT.pl (Zhang *et al*., 2012) with MAFFT as a parameter in protein sequence alignment. For inferring selection at the sublocus in sequences, we split CDS alignments with a 60‐base (20 AAs) window and walking step by 3 bases (1 AA) through the SPLIT tool in KaKs_Calculator. The final phylogenetic tree pictures were made by FigTree (v1.4.3) (Rambaut and Drummond, 2007), and the selective pressure analysis was drawn by the R package ggplot2 (Wickham, 2016).

### Construction of *V. mali* cDNA libraries

The transcript levels of the target genes were measured by qRT‐PCR. *V. mali* was cultured on PDA medium for 3 days. Apple twig tissues of *Malus domestica* Borkh. cv. Fuji were inoculated with *V. mali* mycelium. The inoculated samples (0 h [0 hpi corresponds to mycelium sampled immediately before inoculation], 12, 24, 36, 48, 60, 72, 84 and 96 h post‐inoculation [hpi]) were acquired and rapidly frozen in liquid nitrogen. The total RNA of the mycelium that formed at the lesion border of apple tree bark was extracted using the RNeasy Micro Kit (Qiagen, Shenzhen, China). Quality‐controlled total RNA was used for first strand cDNA synthesis with the RevertAid^TM^ First Strand cDNA Synthesis Kit (Fermentas, Shenzhen, China). The accession numbers of the proteins are listed in Supplementary Table S3.

### Transcript level analysis (qRT‐PCR analysis)

The RNA of the mycelium that formed at the lesion border of apple tree bark was extracted using the RNeasy Micro Kit (Qiagen, Shenzhen, PRC) following its recommended protocol. First strand cDNA was synthesized using the Revert Aid™ First Strand cDNA Synthesis Kit (Fermentas, Shenzhen, China) following the manufacturer's instructions. SYBR green qRT‐PCR assays were performed to quantify relative transcript levels at different time points (12, 24, 36, 48, 60, 72, 84 and 96 h post‐inoculation [hpi]) relative to the expression level at 0 hpi (0 hpi corresponds to mycelium sampled immediately before inoculation). The relative expression levels of each gene were calculated using the 2^−ΔΔCT^ method (Livak and Schmittgen, 2001). *G6PDH* (*glucose‐6‐phosphate dehydrogenase*) of *V. mali* was selected as the housekeeping gene (Yin *et al*., 2013). Primers are given in Supplementary Table S4. Data from three replicates were used to calculate the means and standard error of the mean. The statistical analyses were performed using Student's* t*‐test with the SAS software package (SAS Institute, Cary, NC, USA).

### Secretory function validation of putative N‐terminal signal peptides

The yeast invertase secretion assay was used to test the function of the putative N‐terminal signal peptides of the predicted secreted proteins. The pSUC2 vector containing the predicted signal peptide was transformed into yeast strain YTK12 using the lithium acetate method (Geitz *et al*., 1995). CMD‐W medium (0.08% tryptophan dropout supplement, 2.5% sucrose, 0.65% yeast nitrogen base without amino acids, 0.1% glucose and 2% agar) and YPRAA medium (1% yeast extract, 2% raffinose, 2% peptone and 2 mg/mL antimycin A; raffinose was the only carbohydrate source) were used for selection. The signal peptides of the non‐secreted Mg87 protein from *Magnaporthe oryzae* and secreted Avr1b protein from oomycetes were used as negative and positive controls, respectively (Gu *et al*., 2011; Shan *et al*., 2004). The primers used for the pSUC2 plasmid construction are listed in Supplementary Table S3.

### *Agrobacterium tumefaciens* infiltration assays

*A. tumefaciens* strain GV3101 carrying an expression plasmid was used for transient expression. The strains were cultured on LB medium containing kanamycin (50 μg/mL) and rifampicin (25 μg/mL). The prepared cells were resuspended in 10 mM MgCl_2_ (pH 5.6), and the bacterial suspension was adjusted to an OD600 = 0.5. The upper leaves of 4‐week‐old *N. benthamiana* were selected for transient expression, and bacterial strains containing pGR106 : *VmHEPs *(either with or without signal peptide) were infiltrated with a syringe. After 3 days to 4 days post‐infiltration, symptoms were evaluated and photographed. Each *VmHEP* gene was tested on 5 *N. benthamiana* leaves and each assay was repeated twice. A total of 15 leaves were tested for each gene.

### RT‐PCR analysis

The RNA of transient expression samples and control samples was extracted using the RNAeasy Plant Mini Kit (Qiagen, Shenzhen, China) following the recommended protocol. First strand cDNA was synthesized using the Revert Aid™ First Strand cDNA Synthesis Kit (Fermentas, Shenzhen, China) according to the manufacturer's instructions. For the RT‐PCR reactions, the temperature programme consisted of the initial denaturation at 95 °C for 5 min, 30 cycles of 95 °C for 30 s, 57 °C for 1 min, 72 °C for 1 min and a final cycle of 10 min at 72 °C. *GAPDH *(*N. benthamiana*) was used as a positive control. The final PCR products were purified, and sequencing was performed by Sangon Biotech Organism Technology, Xi'an, China.

### Generation of gene deletion mutants

For target gene deletion, PEG‐mediated homologous recombination gene deletion was performed. The gene deletion cassette with three components used the *hygromycin B phosphotransferase* gene (*hph*) or the *Neo* gene as a selective marker for single gene deletion. Both the *hph* and the *Neo* gene were used as selective markers for double gene deletion. The *hph* gene was amplified with primers HpH‐F and Hph‐R from pBIG2RHPH2, and the *Neo* gene was amplified with primers Neo‐F and Neo‐R. The upstream and downstream flanking sequences were generated with 1F/2R and 3F/4R, respectively (Fig. S6). Then, the upstream‐selected gene‐downstream cassette was generated by double‐joint PCR (Yu *et al*., 2004). The cassette was later transformed into the protoplasts of *V. mali*, and the transferred protoplasts were selected by culturing on medium with the corresponding antibiotic (300 μL/mL geneticin or 120 μL/mL hygromycin) (Gao *et al*., 2011). The obtained putative gene deletion mutants were validated by PCR using 5F and 6R, 7F and Neo(Hph)‐CR, Neo(Hph)‐CF and 8R, and Neo(Hph)‐CF and Neo(Hph)‐CR. 5F and 6R were used to detect the target gene (*VmHEPs*); Neo‐CF and Neo(Hph)‐CR were used to detect the resistant gene (*Neo* or *Hph*), which was used to replace the target gene (*VmHEPs*); 7F and Neo(Hph)‐CR were used to confirm whether the upstream sequence of the introduced resistance gene was fused at the right position; and Neo(Hph)‐CF and 8R were used to confirm if the downstream sequence of the introduced resistance gene was fused at the right position (Fig. S6). Final confirmation of gene deletion was obtained through Southern blot hybridization using the DIG DNA Labeling and Detection Kit II (Roche, Mannheim, Germany) based on the recommended protocol. All the primers used in this section are given in Supplementary Table S5.

### Pathogenicity, conidiation and vegetative growth of mutants

For the pathogenicity assay, detached apple (*M. domestica* Borkh. cv. Fuji) twigs and leaves were prepared and were surface sterilized with 0.6% sodium hypochlorite for 10 min. The pre‐processed twigs and leaves were washed with sterile water three times. The twigs were wounded at three locations using a flat iron (5 mm diameter), and the leaves were wounded at four locations using a sterile needle. The wounded samples were inoculated with mycelium agar (5 mm diameter) obtained from the edge of an activated colony of deletion mutants on PDA. The scabs were measured at 5 dpi. The colony was examined at 3 days for vegetative growth and conidiation measurements, and the pycnidia were counted per square centimetre (cm^−2^) at 40 days and cultured on PDA medium. The data were analysed by Student's *t*‐test using the SAS software package (SAS Institute, Cary, NC, USA).

## Supporting information


**Fig. S1** Gene deletion validation of *VmHEPs* by Polymerase Chain Reaction (PCR) analysis and Southern blot. (A) Confirmation of *VmHEPs* deletion mutants by PCR analysis with four pairs of primers and (B) wild type 03‐8 was as control. 1: 7F/Neo‐CR detected upstream fusion segment. 2: Neo‐CF/8R detected downstream fusion segment. 3: 5F/6R detected targeted gene and 4: Neo‐CF/Neo‐CR detected incoming resistant gene Neo. M: Maker. (C) The further Southern blotting confirmed successful deletion of each *VmHEP *gene*.*

**Fig. S2** Relative expression level of *VmHEP1* in* ∆VmHEP2 *and* VmHEP2* in *∆VmHEP1*, relative to *VmHEP1 *or* VmHEP2 *in wild type 03‐8 on non infection stage. (A) Relative expression level of *VmHEP1* in deletion mutant *∆VmHEP2 *cultured on potato dextrose agar (PDA) was not significantly changed (*P* > 0.05). (B) Relative expression level of *VmHEP2* in deletion mutant* ∆VmHEP1* cultured on PDA was also not significantly changed (*P* > 0.05). Transcript levels of *VmHEP1/2* in wild type 03‐8 cultured on PDA were set to 1 and the *G6PDH *was used as housekeeping gene.

**Fig. S3** Double gene deletion validation by Polymerase Chain Reaction (PCR) analysis. (A) Confirmation of deletion of *VmHEP1*&*VmHEP2* and importation of resistance genes by PCR analysis and (B) wild type 03‐8 was as control.

**Fig. S4** Conidiation and vegetative growth of mutants. (A, B) 03‐8 and *∆VmHEP1*&*VmHEP2* grown on potato dextrose agar (PDA) for 3 days, 25 °C. (C, D) Pycnidia were counted per square centimetre (cm^−2^) cultured on PDA for 40 days, 25 °C.

**Fig. S5** Stress resistance of mutants. (A, B) 03‐8 and *∆VmHEP1*&*VmHEP2* were cultured on PDA supplemented with 0.06% H_2_O_2_ and (C, D) 0.5 M KCl. Pictures were taken after 4 days of incubation at 25 °C.

**Fig. S6** Schematic diagrams of PEG mediated gene deletion. The *hph* gene was amplified with primers HpH‐F and Hph‐R, and the *Neo* gene was amplified with primers Neo‐F and Neo‐R. The upstream and downstream flanking sequences were generated with 1F/2R and 3F/4R, respectively. 5F and 6R were used to detect the target gene (*VmHEPs*); Neo(Hph)‐CF and Neo(Hph)‐CR were used to detect the resistant gene (*Neo* or* Hph*), which was used to replace the target gene (*VmHEPs*); 7F and Neo(Hph)‐CR were used to confirm if the upstream sequence of the imported resistance genes was fused to the right position; Neo(Hph)‐CF and 8R were used to confirm if the downstream sequence of the introduced resistance genes was fused to the right position.

**Table S1** Identified Hce2 in *V. mali* secretome. Searching of *V. mali* secretome against Pfam database using Perl script pfam_scan.pl and proteins with significant hits (e‐value < 1e^−5^) are considered as Hce2 homologues. Ind. E‐value: significance, if this was the only domain that had been identified. Cond. E‐value: significance, if the domain and the query were true homologues.

**Table S2** BLAST table shows the similarity between *V. mali* Hce2s and their Hce2 homologues, non‐significant hits of Hce2 homologues are filtered out. The table was output of BLASTP (protein‐protein BLAST) with e‐value = 1e^−5 ^in table format, *V. mali* Hce2s and all of full‐length sequences (453 proteins) constructing Hce2 HMM model were used to make BLAST database. Query: Names of *V. mali* Hce2s; Subject: Names of *V.mali *Hce2s or Uniprot ID of Hce2 homologues; Query. Start and Query. End: alignment start and end of query sequence; Subject. Start and Subject. End: alignment start and end of subject sequence.

**Table S3** Primers used in pSUC2 plasmid constructs in this study.

**Table S4** Primers for quantitative Reverse Transcription‐Polymerase Chain Reaction (qRT‐PCR) in this study.

**Table S5** Primers for gene deletion and Polymerase Chain Reaction (PCR) analysis in this study.
