# Supplementary figures and images for: Hce2 domain‐containing effectors contribute to the full virulence of Valsa mali in a redundant manner
Source: Mol Plant Pathol. 2019 Mar 26;20(6):843–56. doi: 10.1111/mpp.12796 (PMC6637899; doi:10.1111/mpp.12796)

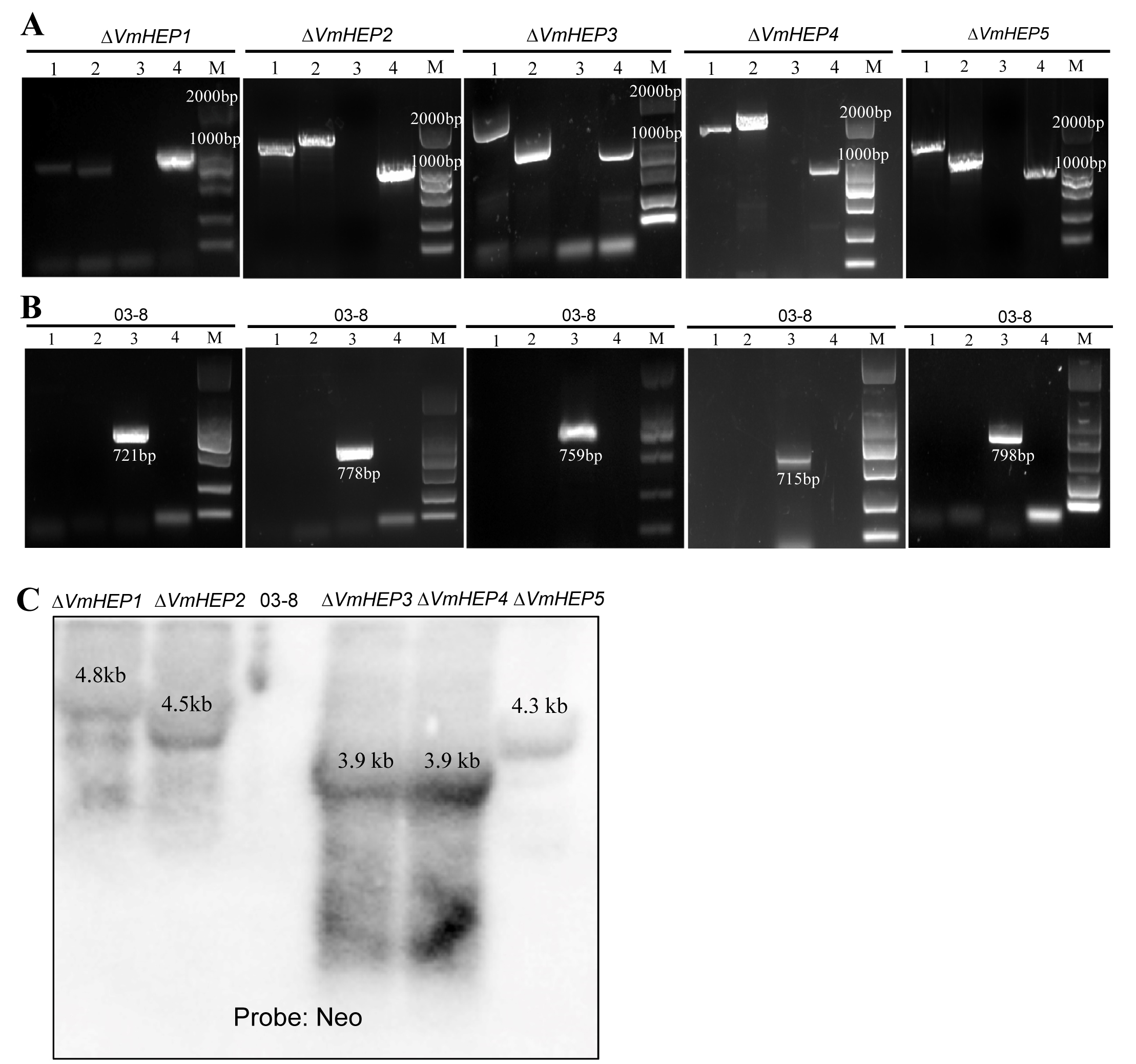

Supplement: Supplementary file 1 — Fig. S1 Gene deletion validation of VmHEPs by Polymerase Chain Reaction (PCR) analysis and Southern blot. (A) Confirmation of VmHEPs deletion mutants by PCR analysis with four pairs of primers and (B) wild type 03‐8 was as control. 1: 7F/Neo‐CR detected upstream fusion segment. 2: Neo‐CF/8R detected downstream fusion segment. 3: 5F/6R detected targeted gene and 4: Neo‐CF/Neo‐CR detected incoming resistant gene Neo. M: Maker. (C) The further Southern blotting confirmed successful deletion of each VmHEP gene. [file MPP-20-843-s001.jpg]

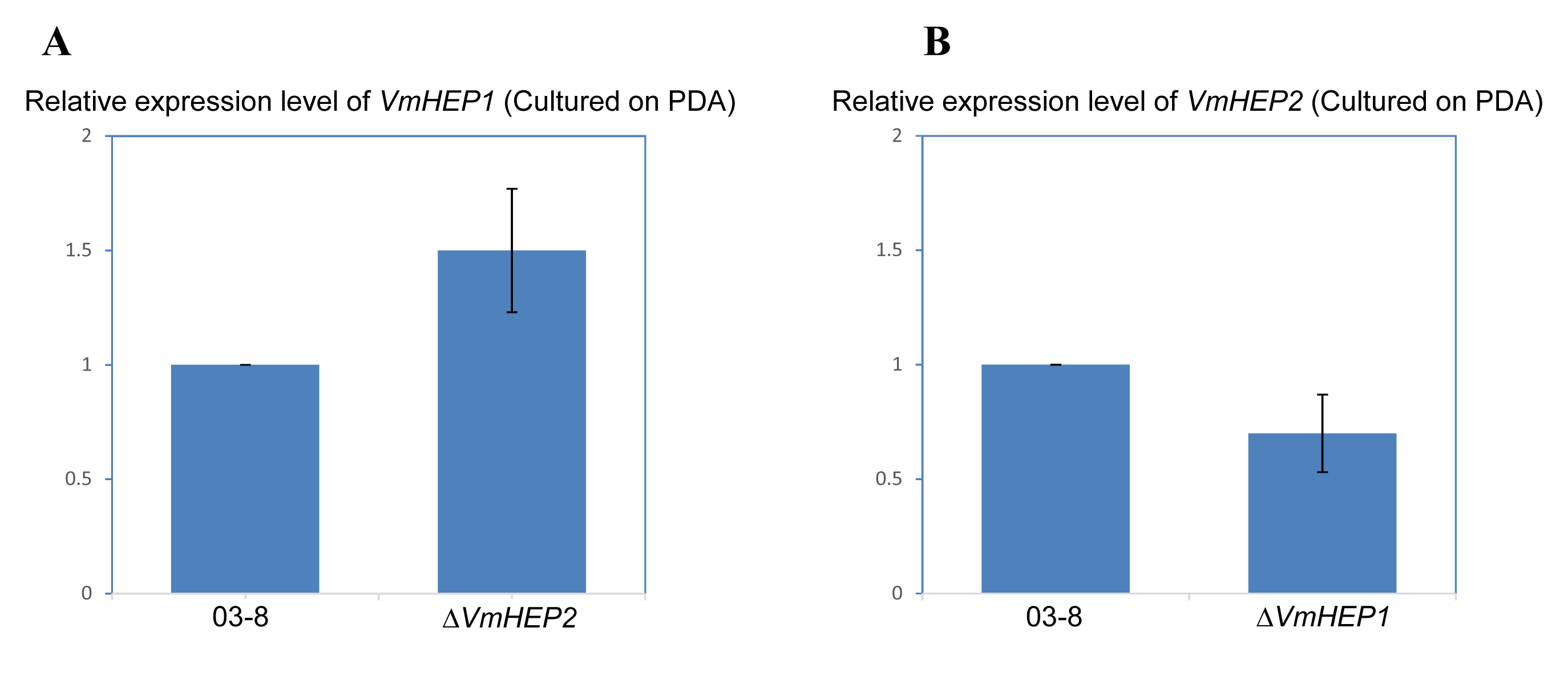

Supplement: Supplementary file 2 — Fig. S2 Relative expression level of VmHEP1 in ∆VmHEP2 and VmHEP2 in ∆VmHEP1, relative to VmHEP1 or VmHEP2 in wild type 03‐8 on non infection stage. (A) Relative expression level of VmHEP1 in deletion mutant ∆VmHEP2 cultured on potato dextrose agar (PDA) was not significantly changed (P > 0.05). (B) Relative expression level of VmHEP2 in deletion mutant ∆VmHEP1 cultured on PDA was also not significantly changed (P > 0.05). Transcript levels of VmHEP1/2 in wild type 03‐8 cultured on PDA were set to 1 and the G6PDH was used as housekeeping gene. [file MPP-20-843-s002.jpg]

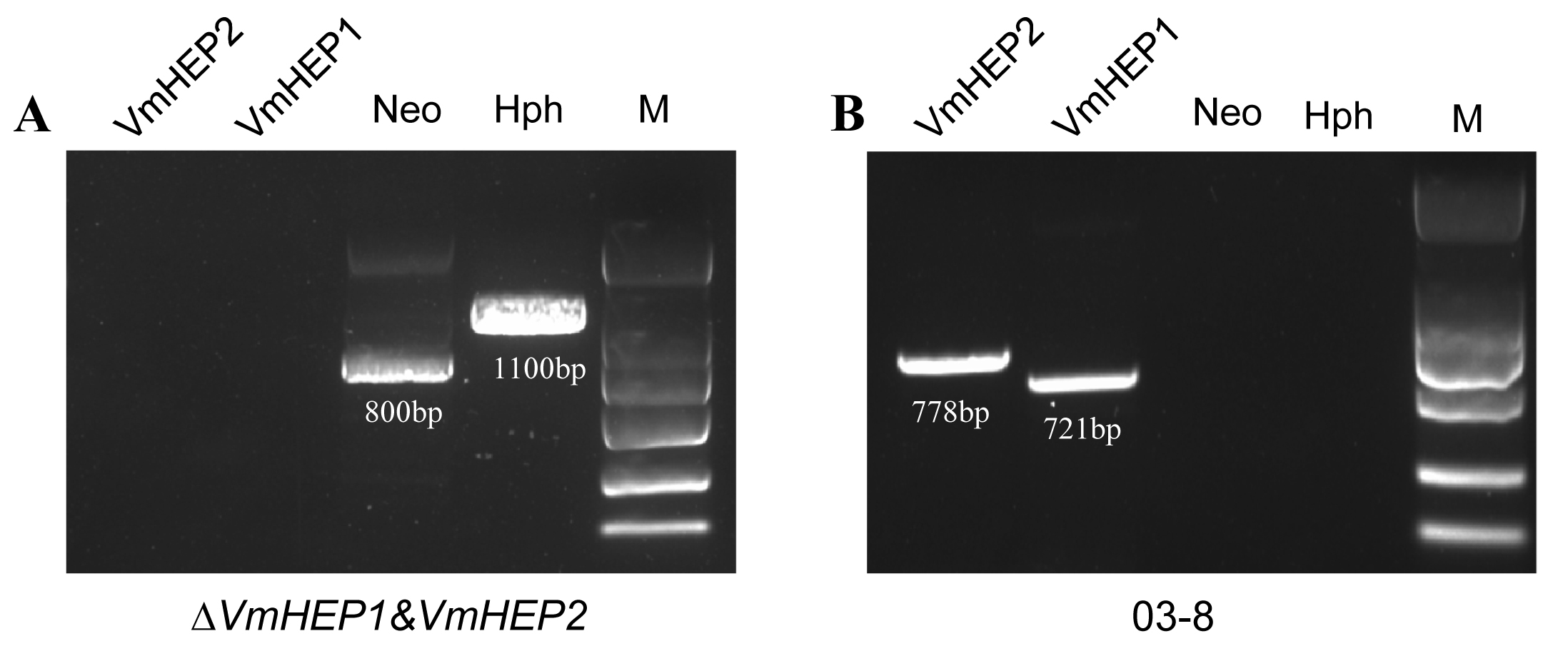

Supplement: Supplementary file 3 — Fig. S3 Double gene deletion validation by Polymerase Chain Reaction (PCR) analysis. (A) Confirmation of deletion of VmHEP1&VmHEP2 and importation of resistance genes by PCR analysis and (B) wild type 03‐8 was as control. [file MPP-20-843-s003.jpg]

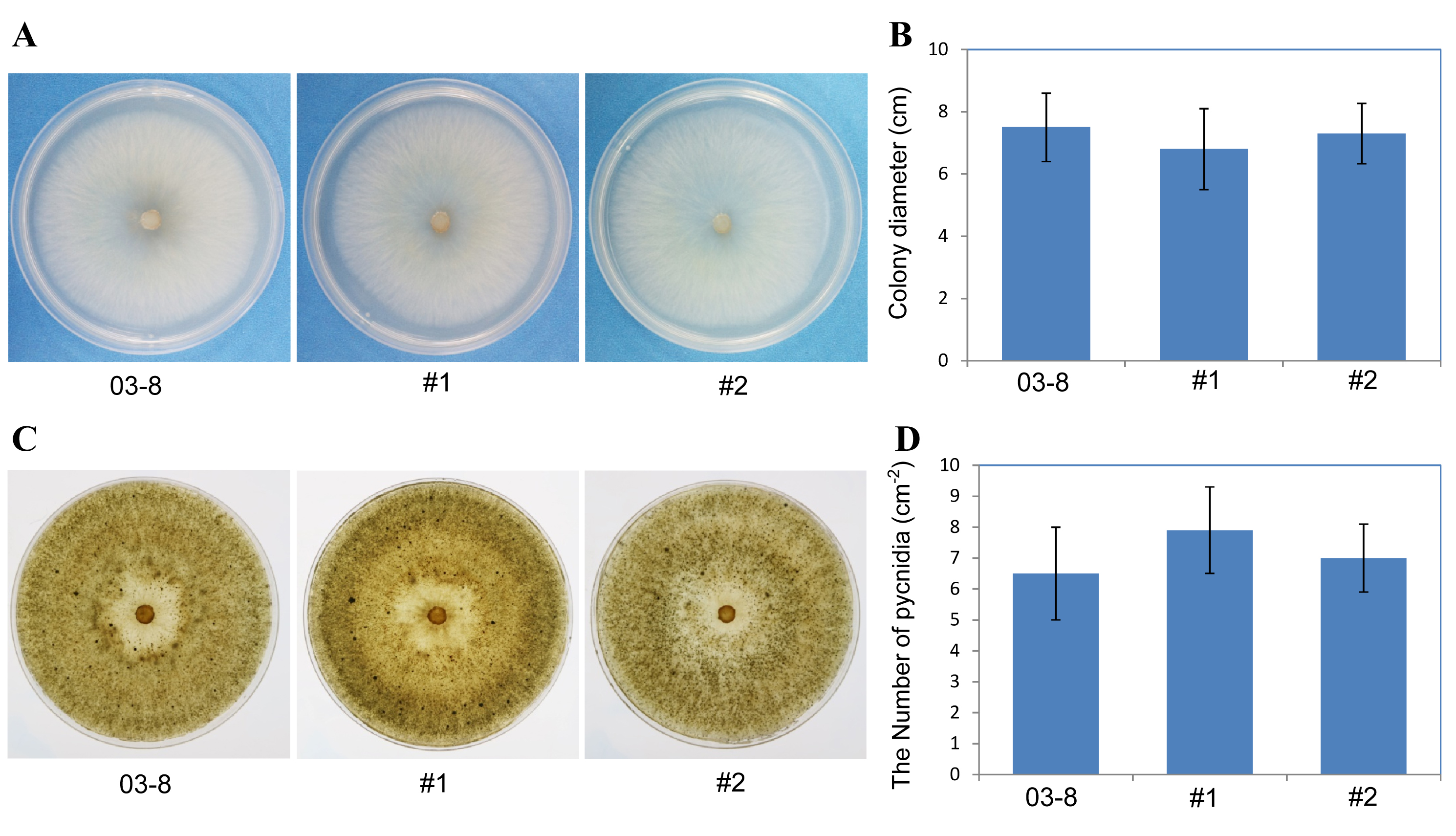

Supplement: Supplementary file 4 — Fig. S4 Conidiation and vegetative growth of mutants. (A, B) 03‐8 and ∆VmHEP1&VmHEP2 grown on potato dextrose agar (PDA) for 3 days, 25 °C. (C, D) Pycnidia were counted per square centimetre (cm−2) cultured on PDA for 40 days, 25 °C. [file MPP-20-843-s004.jpg]

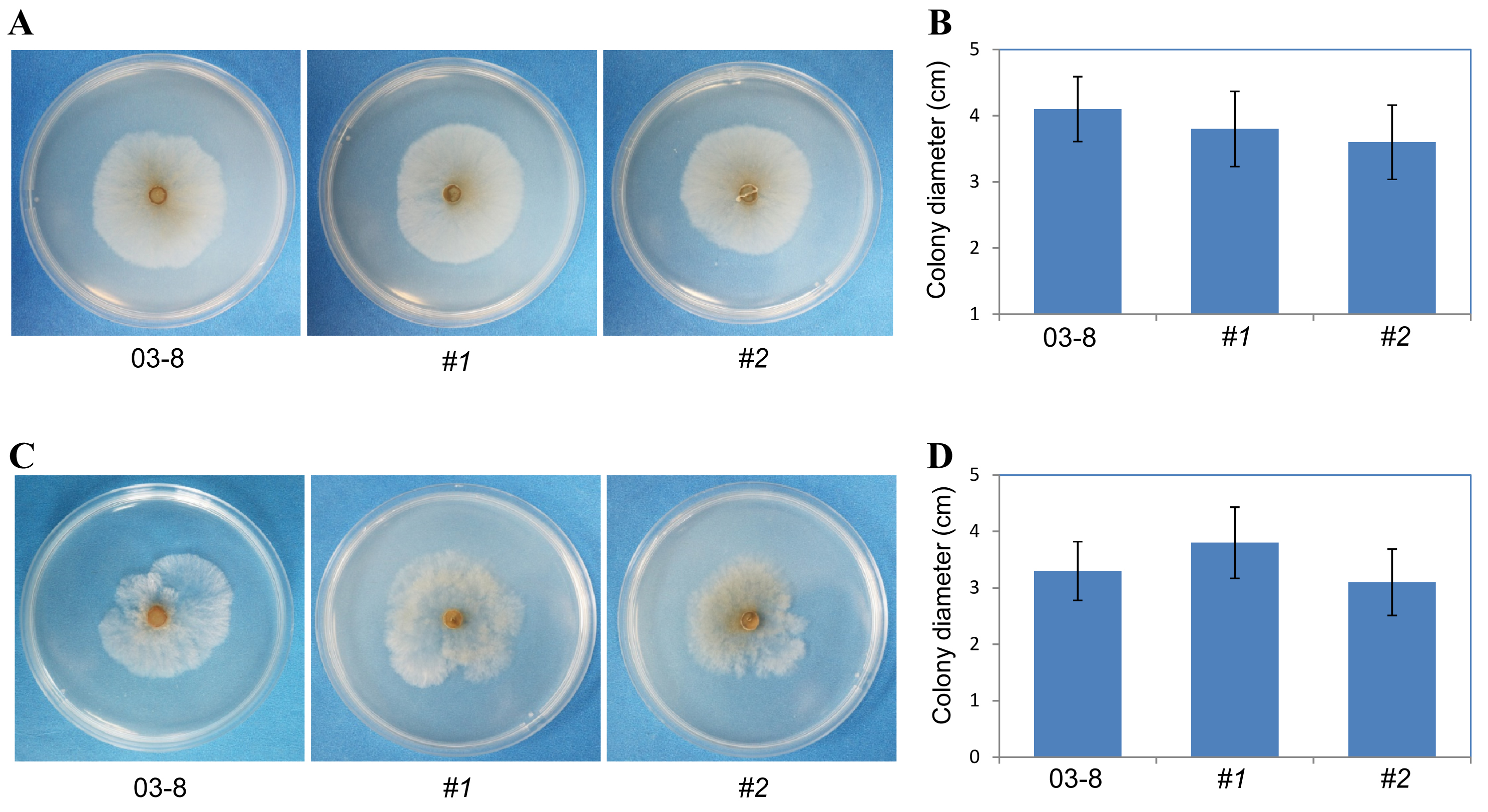

Supplement: Supplementary file 5 — Fig. S5 Stress resistance of mutants. (A, B) 03‐8 and ∆VmHEP1&VmHEP2 were cultured on PDA supplemented with 0.06% H2O2 and (C, D) 0.5 M KCl. Pictures were taken after 4 days of incubation at 25 °C. [file MPP-20-843-s005.jpg]

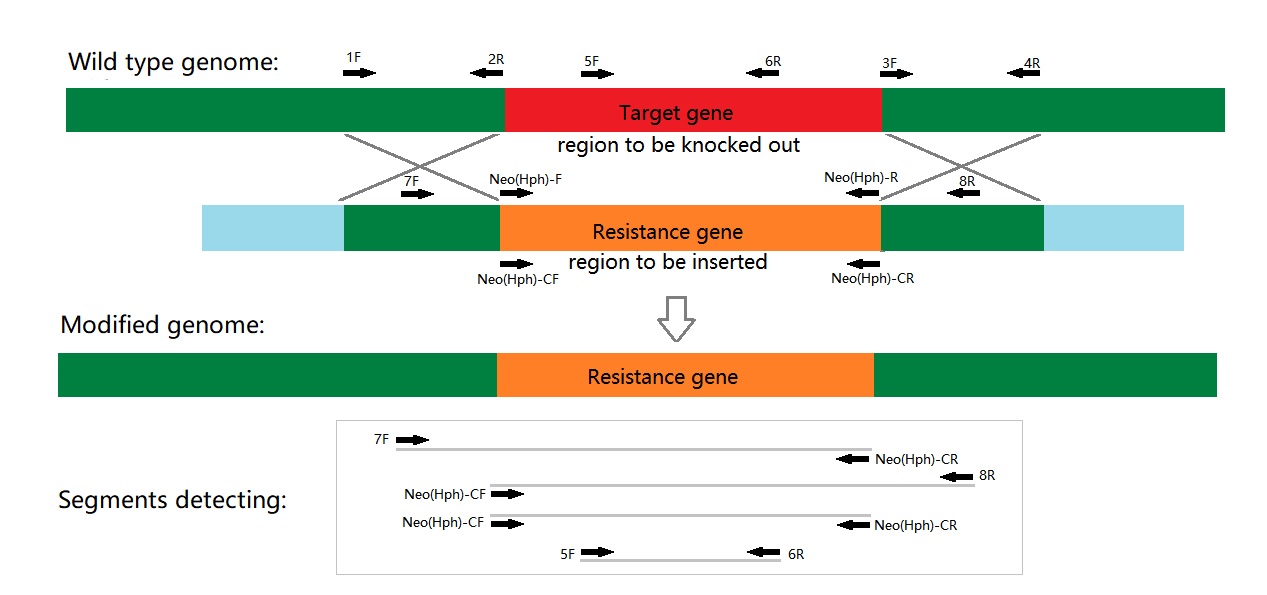

Supplement: Supplementary file 6 — Fig. S6 Schematic diagrams of PEG mediated gene deletion. The hph gene was amplified with primers HpH‐F and Hph‐R, and the Neo gene was amplified with primers Neo‐F and Neo‐R. The upstream and downstream flanking sequences were generated with 1F/2R and 3F/4R, respectively. 5F and 6R were used to detect the target gene (VmHEPs); Neo(Hph)‐CF and Neo(Hph)‐CR were used to detect the resistant gene (Neo or Hph), which was used to replace the target gene (VmHEPs); 7F and Neo(Hph)‐CR were used to confirm if the upstream sequence of the imported resistance genes was fused to the right position; Neo(Hph)‐CF and 8R were used to confirm if the downstream sequence of the introduced resistance genes was fused to the right position. [file MPP-20-843-s006.jpg]
