# Supplementary material for: Hce2 domain‐containing effectors contribute to the full virulence of Valsa mali in a redundant manner
Source: Mol Plant Pathol. 2019 Mar 26;20(6):843–56. doi: 10.1111/mpp.12796 (PMC6637899; doi:10.1111/mpp.12796)
Supplement: Supplementary file 7 — Table S1 Identified Hce2 in V. mali secretome. Searching of V. mali secretome against Pfam database using Perl script pfam_scan.pl and proteins with significant hits (e‐value < 1e−5) are considered as Hce2 homologues. Ind. E‐value: significance, if this was the only domain that had been identified. Cond. E‐value: significance, if the domain and the query were true homologues. [file MPP-20-843-s007.docx]

|  | | | | | | | | | | | |
| --- | --- | --- | --- | --- | --- | --- | --- | --- | --- | --- | --- |
| Id | Length | Alignment Start | Alignment End | HMM Start | HMM End | HMM Name | HMM Accession | HMM Length | Bit Score | Ind. E-value | Cond. E-value |
| Vm1g_09394  (VmHEP1) | 179 | 67 | 158 | 17 | 102 | Hce2 | PF14856.6 | 102 | 38.2 | 1.4e-09 | 7.6e-14 |
| Vm1g_09395  (VmHEP2) | 181 | 46 | 153 | 2 | 102 | Hce2 | PF14856.6 | 102 | 51.32 | 1.1e-13 | 6.2e-18 |
| Vm1g_00980  (VmHEP5) | 189 | 73 | 171 | 2 | 102 | Hce2 | PF14856.6 | 102 | 54.95 | 8.2e-15 | 4.6e-19 |
| Vm1g_07403  (VmHEP4) | 196 | 62 | 170 | 2 | 102 | Hce2 | PF14856.6 | 102 | 49.1 | 5.4e-13 | 3e-17 |
| Vm1g_05547  (VmHEP3) | 213 | 10 | 152 | 10 | 102 | Hce2 | PF14856.6 | 102 | 52.15 | 6.1e-14 | 3.4e-18 |
| Table S1. Identified Hce2 in *V. mali* secretome. Searching of *V. mali* secretome against Pfam database using Perl script pfam_scan.pl and proteins with significant hits (e-value < 1e-5) are considered as Hce2 homologs. Ind. E-value: significance, if this is the only domain that had been identified. Cond. E-value: significance, if the domain and the query are true homologs. | | | | | | | | | | | |
