# Supplementary material for: Hce2 domain‐containing effectors contribute to the full virulence of Valsa mali in a redundant manner
Source: Mol Plant Pathol. 2019 Mar 26;20(6):843–56. doi: 10.1111/mpp.12796 (PMC6637899; doi:10.1111/mpp.12796)
Supplement: Supplementary file 8 — Table S2 BLAST table shows the similarity between V. mali Hce2s and their Hce2 homologues, non‐significant hits of Hce2 homologues are filtered out. The table was output of BLASTP (protein‐protein BLAST) with e‐value = 1e−5 in table format, V. mali Hce2s and all of full‐length sequences (453 proteins) constructing Hce2 HMM model were used to make BLAST database. Query: Names of V. mali Hce2s; Subject: Names of V.mali Hce2s or Uniprot ID of Hce2 homologues; Query. Start and Query. End: alignment start and end of query sequence; Subject. Start and Subject. End: alignment start and end of subject sequence. [file MPP-20-843-s008.docx]

| Similarity between *V. mali* Hce2s and their Hce2 homologs | | | | | | | | | | | |
| --- | --- | --- | --- | --- | --- | --- | --- | --- | --- | --- | --- |
| Query | Subject | % identity | Alignment length | Mismatches | Gap opens | Q. Start | Q. End | S. Start | S. End | Evalue | Bit score |
| VmHEP1 | VmHEP2 | 35.68 | 185 | 100 | 8 | 1 | 176 | 1 | 175 | 4.00E-29 | 97.4 |
| VmHEP1 | VmHEP5 | 29.59 | 98 | 57 | 2 | 78 | 175 | 103 | 188 | 2.00E-12 | 50.8 |
| VmHEP1 | VmHEP4 | 25.21 | 119 | 73 | 5 | 66 | 175 | 76 | 187 | 2.00E-06 | 33.9 |
| VmHEP1 | VmHEP3 | 22.22 | 81 | 58 | 3 | 96 | 173 | 90 | 168 | 0.009 | 23.1 |
| VmHEP2 | VmHEP5 | 28.7 | 115 | 63 | 4 | 45 | 154 | 72 | 172 | 2.00E-09 | 42.7 |
| VmHEP2 | VmHEP4 | 25.22 | 115 | 77 | 4 | 44 | 153 | 60 | 170 | 0.000000007 | 41.2 |
| VmHEP2 | VmHEP3 | 26.53 | 98 | 60 | 6 | 59 | 153 | 64 | 152 | 0.0005 | 26.9 |
| VmHEP5 | VmHEP3 | 30.86 | 81 | 52 | 2 | 98 | 176 | 79 | 157 | 1E-10 | 46.6 |
| VmHEP5 | VmHEP4 | 30.48 | 105 | 59 | 6 | 92 | 185 | 83 | 184 | 0.0000002 | 37.4 |
| VmHEP4 | VmHEP3 | 30.97 | 155 | 91 | 9 | 45 | 196 | 33 | 174 | 4E-14 | 56.2 |
| VmHEP1 | A0A1C1XMX1_9PEZI | 33.12 | 154 | 91 | 6 | 28 | 176 | 23 | 169 | 5.00E-21 | 82 |
| VmHEP1 | A0A063C3L5_9HYPO | 33.33 | 108 | 60 | 5 | 70 | 175 | 63 | 160 | 4.00E-08 | 45.4 |
| VmHEP1 | A0A1L9SH47_9EURO | 31.71 | 123 | 66 | 6 | 53 | 175 | 28 | 132 | 1.00E-07 | 43.5 |
| VmHEP1 | A0A1J7IPV1_9PEZI | 35.53 | 76 | 44 | 4 | 101 | 175 | 90 | 161 | 4.00E-07 | 42.7 |
| VmHEP1 | A0A0B4H057_9HYPO | 30.48 | 105 | 65 | 4 | 71 | 175 | 38 | 134 | 5.00E-07 | 42 |
| VmHEP1 | A0A0G2HFC0_9PEZI | 28.46 | 123 | 76 | 5 | 53 | 172 | 72 | 185 | 1.00E-06 | 41.6 |
| VmHEP1 | A0A168E924_9HYPO | 28.89 | 90 | 56 | 4 | 71 | 160 | 68 | 149 | 2.00E-06 | 41.2 |
| VmHEP1 | A0A0B4IF08_9HYPO | 29.52 | 105 | 66 | 4 | 71 | 175 | 38 | 134 | 2.00E-06 | 40 |
| VmHEP1 | A0A0L0N3K3_9HYPO | 25.78 | 128 | 85 | 4 | 50 | 177 | 53 | 170 | 2.00E-06 | 40.8 |
| VmHEP1 | E9EQD3_METRA | 28.7 | 108 | 65 | 4 | 70 | 175 | 37 | 134 | 3.00E-06 | 39.7 |
| VmHEP1 | A0A1J7JJP9_9PEZI | 31.9 | 116 | 59 | 7 | 68 | 175 | 62 | 165 | 3.00E-06 | 40.4 |
| VmHEP1 | A0A0D9P6B2_METAN | 28.7 | 108 | 65 | 4 | 70 | 175 | 37 | 134 | 3.00E-06 | 39.7 |
| VmHEP1 | E9EFB2_METAQ | 28.3 | 106 | 68 | 4 | 70 | 175 | 63 | 160 | 7.00E-06 | 39.7 |
| VmHEP1 | G1X0T5_ARTOA | 30.56 | 108 | 63 | 4 | 53 | 158 | 1354 | 1451 | 8.00E-06 | 40 |
| VmHEP1 | A0A231M6V3_9EURO | 27.59 | 174 | 101 | 8 | 1 | 172 | 1 | 151 | 9.00E-06 | 38.9 |
| VmHEP2 | A0A1C1XMX1_9PEZI | 35.63 | 174 | 99 | 7 | 8 | 175 | 3 | 169 | 7.00E-25 | 92.8 |
| VmHEP2 | A0A231MFM4_9EURO | 38.46 | 91 | 45 | 4 | 46 | 133 | 1018 | 1100 | 5.00E-09 | 49.7 |
| VmHEP2 | A0A1J7JJP9_9PEZI | 27.89 | 147 | 87 | 7 | 29 | 173 | 35 | 164 | 8.00E-09 | 47.4 |
| VmHEP2 | A0A0A2KZJ1_PENIT | 36.52 | 115 | 51 | 6 | 46 | 153 | 942 | 1041 | 1.00E-08 | 48.5 |
| VmHEP2 | F2PXS5_TRIEC | 30.08 | 133 | 73 | 7 | 42 | 171 | 1079 | 1194 | 2.00E-07 | 44.7 |
| VmHEP2 | A0A084FYT1_9PEZI | 29.09 | 110 | 67 | 6 | 46 | 154 | 1352 | 1451 | 2.00E-07 | 44.7 |
| VmHEP2 | B6HA46_PENRW | 26.28 | 137 | 84 | 7 | 39 | 173 | 1322 | 1443 | 3.00E-07 | 44.3 |
| VmHEP2 | W6Q8K4_PENRF | 26.28 | 137 | 84 | 7 | 39 | 173 | 1348 | 1469 | 4.00E-07 | 44.3 |
| VmHEP2 | A0A1L9TK74_9EURO | 27.69 | 130 | 81 | 5 | 39 | 165 | 1357 | 1476 | 5.00E-07 | 43.9 |
| VmHEP2 | X0J6P9_FUSOX | 33.06 | 121 | 64 | 6 | 17 | 134 | 10 | 116 | 5.00E-07 | 42.4 |
| VmHEP2 | N1S439_FUSC4 | 33.06 | 121 | 64 | 6 | 17 | 134 | 10 | 116 | 5.00E-07 | 42.4 |
| VmHEP2 | A0A1Y1ZQW6_9PLEO | 31.2 | 125 | 70 | 5 | 49 | 171 | 39 | 149 | 5.00E-07 | 42.4 |
| VmHEP2 | D4APW5_ARTBC | 32.54 | 126 | 72 | 5 | 42 | 165 | 967 | 1081 | 6.00E-07 | 43.5 |
| VmHEP2 | R0KMA8_SETT2 | 31.48 | 108 | 54 | 5 | 43 | 145 | 33 | 125 | 7.00E-07 | 41.6 |
| VmHEP2 | A0A1Y1ZRJ4_9PLEO | 29.37 | 126 | 69 | 6 | 35 | 155 | 28 | 138 | 1.00E-06 | 41.6 |
| VmHEP2 | X0BXX1_FUSOX | 34.29 | 105 | 54 | 5 | 31 | 134 | 26 | 116 | 1.00E-06 | 41.2 |
| VmHEP2 | N4TX09_FUSC1 | 33.06 | 121 | 64 | 6 | 17 | 134 | 10 | 116 | 1.00E-06 | 41.2 |
| VmHEP2 | W9I5G2_FUSOX | 34.29 | 105 | 54 | 5 | 31 | 134 | 26 | 116 | 1.00E-06 | 41.2 |
| VmHEP2 | A0A2H3GVP1_FUSOX | 34.29 | 105 | 54 | 5 | 31 | 134 | 26 | 116 | 1.00E-06 | 41.2 |
| VmHEP2 | A0A2H3SQC4_FUSOX | 34.29 | 105 | 54 | 5 | 31 | 134 | 26 | 116 | 1.00E-06 | 41.2 |
| VmHEP2 | A0A0D2XLE6_FUSO4 | 34.29 | 105 | 54 | 5 | 31 | 134 | 26 | 116 | 1.00E-06 | 41.2 |
| VmHEP2 | F2ST22_TRIRC | 33.06 | 124 | 66 | 6 | 46 | 165 | 1360 | 1470 | 2.00E-06 | 42 |
| VmHEP2 | A0A2B7WF69_9EURO | 34.74 | 95 | 49 | 5 | 61 | 153 | 109 | 192 | 2.00E-06 | 41.6 |
| VmHEP2 | A0A1V6TRA6_9EURO | 29.31 | 116 | 72 | 5 | 39 | 153 | 1349 | 1455 | 2.00E-06 | 42 |
| VmHEP2 | A0A1J7IPV1_9PEZI | 29.46 | 129 | 75 | 5 | 40 | 165 | 42 | 157 | 3.00E-06 | 40.4 |
| VmHEP2 | K9GQZ3_PEND2 | 34.82 | 112 | 61 | 6 | 54 | 165 | 1409 | 1508 | 3.00E-06 | 41.6 |
| VmHEP2 | A0A175W6R6_9PEZI | 26.72 | 116 | 75 | 5 | 39 | 153 | 1343 | 1449 | 5.00E-06 | 40.8 |
| VmHEP2 | A0A0B4I3M8_9HYPO | 32.03 | 128 | 69 | 6 | 35 | 161 | 33 | 143 | 5.00E-06 | 39.7 |
| VmHEP2 | A0A1R3RHQ9_ASPC5 | 33.05 | 118 | 62 | 6 | 54 | 171 | 1409 | 1509 | 5.00E-06 | 40.8 |
| VmHEP2 | Q7S8L7_NEUCR | 35.16 | 91 | 48 | 4 | 46 | 133 | 1371 | 1453 | 6.00E-06 | 40.4 |
| VmHEP2 | G4UIT9_NEUT9 | 35.16 | 91 | 48 | 4 | 46 | 133 | 1371 | 1453 | 6.00E-06 | 40.4 |
| VmHEP2 | A0A0G2HFC0_9PEZI | 30.08 | 133 | 74 | 8 | 45 | 173 | 70 | 187 | 8.00E-06 | 39.3 |
| VmHEP2 | A0A1J7IKZ9_9PEZI | 38.03 | 71 | 37 | 3 | 83 | 153 | 88 | 151 | 8.00E-06 | 39.3 |
| VmHEP2 | F7WCI4_SORMK | 36.67 | 90 | 48 | 4 | 46 | 134 | 1342 | 1423 | 9.00E-06 | 40 |
| VmHEP5 | A0A0G2HFC0_9PEZI | 52.6 | 192 | 83 | 7 | 1 | 188 | 1 | 188 | 5.00E-63 | 192 |
| VmHEP5 | A0A1C1WQP7_9PEZI | 51.83 | 191 | 83 | 8 | 1 | 186 | 1 | 187 | 1.00E-59 | 183 |
| VmHEP5 | A0A1Y2DR20_9PEZI | 28.12 | 160 | 98 | 6 | 31 | 186 | 39 | 185 | 1.00E-12 | 58.2 |
| VmHEP5 | T5ACM1_OPHSC | 30.95 | 126 | 75 | 5 | 63 | 186 | 62 | 177 | 2.00E-12 | 57.8 |
| VmHEP5 | A0A1C1XK81_9PEZI | 27.74 | 137 | 92 | 5 | 55 | 187 | 43 | 176 | 4.00E-12 | 56.6 |
| VmHEP5 | A0A231M6V3_9EURO | 33.61 | 119 | 69 | 5 | 71 | 186 | 41 | 152 | 1.00E-11 | 55.1 |
| VmHEP5 | W6PUK1_PENRF | 30 | 140 | 79 | 7 | 52 | 187 | 25 | 149 | 1.00E-11 | 54.7 |
| VmHEP5 | A0A0G4PXZ2_PENCA | 30 | 140 | 79 | 7 | 52 | 187 | 25 | 149 | 1.00E-11 | 54.7 |
| VmHEP5 | T0LDB1_COLGC | 30.83 | 120 | 77 | 4 | 71 | 185 | 93 | 211 | 3.00E-10 | 52.4 |
| VmHEP5 | W6YYG6_COCMI | 30.89 | 123 | 72 | 6 | 67 | 187 | 30 | 141 | 3.00E-10 | 50.8 |
| VmHEP5 | L2GJJ2_COLGN | 30.83 | 120 | 77 | 4 | 71 | 185 | 84 | 202 | 4.00E-10 | 52 |
| VmHEP5 | A0A1Y1ZRJ4_9PLEO | 33.61 | 122 | 65 | 6 | 71 | 185 | 37 | 149 | 1.00E-09 | 49.3 |
| VmHEP5 | A0A0K8LG13_9EURO | 32.5 | 120 | 71 | 5 | 71 | 187 | 48 | 160 | 2.00E-09 | 48.9 |
| VmHEP5 | G9NWZ1_HYPAI | 26.02 | 123 | 81 | 4 | 69 | 186 | 37 | 154 | 4.00E-09 | 48.1 |
| VmHEP5 | A0A0W7W133_9HYPO | 26.02 | 123 | 81 | 4 | 69 | 186 | 37 | 154 | 7.00E-09 | 47.4 |
| VmHEP5 | A0A1L9SH47_9EURO | 30.25 | 119 | 69 | 6 | 71 | 187 | 25 | 131 | 9.00E-09 | 47 |
| VmHEP5 | D4B299_ARTBC | 26.89 | 119 | 79 | 4 | 71 | 187 | 133 | 245 | 1.00E-08 | 48.5 |
| VmHEP5 | A0A1C1XMX1_9PEZI | 34.38 | 64 | 40 | 1 | 124 | 185 | 102 | 165 | 1.00E-08 | 47.4 |
| VmHEP5 | A0A084FYT1_9PEZI | 32.54 | 126 | 62 | 7 | 73 | 186 | 1352 | 1466 | 1.00E-08 | 48.9 |
| VmHEP5 | X0J6P9_FUSOX | 29.77 | 131 | 79 | 6 | 63 | 187 | 27 | 150 | 1.00E-08 | 47 |
| VmHEP5 | N1S439_FUSC4 | 29.77 | 131 | 79 | 6 | 63 | 187 | 27 | 150 | 1.00E-08 | 47 |
| VmHEP5 | W9I5G2_FUSOX | 29.77 | 131 | 79 | 6 | 63 | 187 | 27 | 150 | 1.00E-08 | 47 |
| VmHEP5 | N4TX09_FUSC1 | 29.77 | 131 | 79 | 6 | 63 | 187 | 27 | 150 | 1.00E-08 | 47 |
| VmHEP5 | A0A2H3GVP1_FUSOX | 29.77 | 131 | 79 | 6 | 63 | 187 | 27 | 150 | 1.00E-08 | 47 |
| VmHEP5 | A0A2H3SQC4_FUSOX | 29.77 | 131 | 79 | 6 | 63 | 187 | 27 | 150 | 1.00E-08 | 47 |
| VmHEP5 | A0A0D2XLE6_FUSO4 | 29.77 | 131 | 79 | 6 | 63 | 187 | 27 | 150 | 1.00E-08 | 46.6 |
| VmHEP5 | M1WDX5_CLAP2 | 26.72 | 131 | 72 | 4 | 67 | 185 | 39 | 157 | 2.00E-08 | 46.6 |
| VmHEP5 | A0A059IXX6_9EURO | 25 | 120 | 82 | 4 | 71 | 188 | 54 | 167 | 2.00E-08 | 46.6 |
| VmHEP5 | A0A0B4I3M8_9HYPO | 28.57 | 126 | 83 | 5 | 64 | 187 | 31 | 151 | 2.00E-08 | 46.2 |
| VmHEP5 | X0BXX1_FUSOX | 32.65 | 98 | 56 | 4 | 63 | 156 | 27 | 118 | 2.00E-08 | 46.2 |
| VmHEP5 | A0A1Q8S5W8_9PEZI | 35.59 | 118 | 69 | 6 | 73 | 186 | 1339 | 1453 | 2.00E-08 | 48.1 |
| VmHEP5 | S0DZK6_GIBF5 | 29.01 | 131 | 80 | 6 | 63 | 187 | 27 | 150 | 2.00E-08 | 46.2 |
| VmHEP5 | A0A229XAE4_9EURO | 32.26 | 124 | 70 | 8 | 70 | 186 | 893 | 1009 | 3.00E-08 | 47.4 |
| VmHEP5 | F2SYY8_TRIRC | 25.83 | 120 | 80 | 5 | 71 | 187 | 58 | 171 | 4.00E-08 | 45.8 |
| VmHEP5 | A0A1C1WX61_9PEZI | 34.21 | 76 | 48 | 2 | 98 | 171 | 84 | 159 | 4.00E-08 | 46.6 |
| VmHEP5 | G0RVY5_HYPJQ | 25 | 76 | 56 | 1 | 113 | 188 | 84 | 158 | 4.00E-08 | 45.4 |
| VmHEP5 | D4ARL0_ARTBC | 25.83 | 120 | 80 | 5 | 71 | 187 | 53 | 166 | 5.00E-08 | 45.4 |
| VmHEP5 | R0KMA8_SETT2 | 27.64 | 123 | 72 | 6 | 67 | 187 | 30 | 137 | 6.00E-08 | 44.7 |
| VmHEP5 | W7LRN6_GIBM7 | 29.77 | 131 | 79 | 6 | 63 | 187 | 18 | 141 | 7.00E-08 | 44.7 |
| VmHEP5 | A0A178B6R9_9PLEO | 31.09 | 119 | 67 | 6 | 70 | 187 | 32 | 136 | 7.00E-08 | 44.7 |
| VmHEP5 | W3WZM5_PESFW | 28 | 125 | 74 | 5 | 80 | 188 | 111 | 235 | 1.00E-07 | 45.1 |
| VmHEP5 | A0A231MA91_9EURO | 31.45 | 124 | 71 | 8 | 70 | 186 | 1351 | 1467 | 2.00E-07 | 45.4 |
| VmHEP5 | W6QDF9_PENRF | 27.08 | 144 | 80 | 9 | 51 | 187 | 24 | 149 | 2.00E-07 | 43.5 |
| VmHEP5 | A0A168E924_9HYPO | 22.05 | 127 | 96 | 3 | 60 | 185 | 37 | 161 | 2.00E-07 | 43.9 |
| VmHEP5 | G3YD37_ASPNA | 30.65 | 124 | 72 | 8 | 70 | 186 | 495 | 611 | 2.00E-07 | 45.1 |
| VmHEP5 | A0A2H2Z448_9HYPO | 23.68 | 76 | 57 | 1 | 113 | 188 | 84 | 158 | 2.00E-07 | 43.5 |
| VmHEP5 | E9ESD5_METRA | 28.12 | 128 | 81 | 6 | 64 | 187 | 31 | 151 | 3.00E-07 | 43.1 |
| VmHEP5 | I1S3E0_GIBZE | 26.72 | 131 | 83 | 6 | 63 | 187 | 29 | 152 | 4.00E-07 | 42.7 |
| VmHEP5 | A0A1L9V1W8_9EURO | 30.65 | 124 | 72 | 8 | 70 | 186 | 1351 | 1467 | 6.00E-07 | 43.9 |
| VmHEP5 | A0A2C5XVA8_9HYPO | 30 | 120 | 72 | 6 | 71 | 187 | 61 | 171 | 7.00E-07 | 42.4 |
| VmHEP5 | A0A162KFR1_CORDF | 25.15 | 171 | 116 | 8 | 21 | 186 | 1323 | 1486 | 9.00E-07 | 43.1 |
| VmHEP5 | A0A0M8N7A2_9HYPO | 34.04 | 47 | 30 | 1 | 140 | 186 | 135 | 180 | 1.00E-06 | 42.4 |
| VmHEP5 | A0A0G4PZ75_PENCA | 30.4 | 125 | 71 | 7 | 70 | 186 | 1352 | 1468 | 2.00E-06 | 42.4 |
| VmHEP5 | A0A0L0N3K3_9HYPO | 28 | 75 | 53 | 1 | 111 | 185 | 92 | 165 | 2.00E-06 | 41.2 |
| VmHEP5 | G9MED9_HYPVG | 25.32 | 79 | 56 | 2 | 110 | 186 | 77 | 154 | 3.00E-06 | 40.4 |
| VmHEP5 | A0A0B4H057_9HYPO | 22.5 | 120 | 91 | 2 | 67 | 185 | 13 | 131 | 3.00E-06 | 40 |
| VmHEP5 | A0A0G4N4A0_9PEZI | 24.26 | 136 | 86 | 5 | 67 | 186 | 120 | 254 | 3.00E-06 | 41.2 |
| VmHEP5 | Q5B824_EMENI | 31.09 | 119 | 71 | 7 | 73 | 185 | 482 | 595 | 3.00E-06 | 41.6 |
| VmHEP5 | A0A151GVI9_9HYPO | 25 | 128 | 84 | 4 | 69 | 185 | 38 | 164 | 4.00E-06 | 40 |
| VmHEP5 | E9EQD3_METRA | 21.14 | 123 | 95 | 2 | 64 | 185 | 10 | 131 | 5.00E-06 | 39.3 |
| VmHEP5 | M3B3B1_PSEFD | 34.44 | 90 | 48 | 4 | 71 | 158 | 1 | 81 | 6.00E-06 | 38.5 |
| VmHEP5 | D4APW5_ARTBC | 29.06 | 117 | 76 | 6 | 73 | 186 | 971 | 1083 | 6.00E-06 | 40.8 |
| VmHEP5 | A0A0D9P6B2_METAN | 21.67 | 120 | 92 | 2 | 67 | 185 | 13 | 131 | 6.00E-06 | 38.9 |
| VmHEP5 | Q2GUK0_CHAGB | 27.78 | 126 | 81 | 6 | 65 | 187 | 27 | 145 | 8.00E-06 | 38.9 |
| VmHEP5 | S8BJU9_DACHA | 29.75 | 121 | 78 | 6 | 70 | 186 | 1355 | 1472 | 9.00E-06 | 40.4 |
| VmHEP5 | A0A010RNY1_9PEZI | 29.52 | 105 | 66 | 5 | 86 | 185 | 116 | 217 | 1.00E-05 | 39.7 |
| VmHEP5 | W6PTM6_PENRF | 26.56 | 128 | 64 | 5 | 63 | 188 | 2 | 101 | 1.00E-05 | 38.1 |
| VmHEP4 | M7SWH3_EUTLA | 27.03 | 111 | 69 | 3 | 62 | 170 | 84 | 184 | 1.00E-08 | 47.8 |
| VmHEP4 | W3XDJ6_PESFW | 34.31 | 102 | 55 | 3 | 71 | 170 | 89 | 180 | 1.00E-08 | 47.8 |
| VmHEP4 | A0A1C1X4S8_9PEZI | 35.85 | 53 | 33 | 1 | 76 | 127 | 75 | 127 | 3.00E-08 | 45.4 |
| VmHEP4 | A0A1C1XMX1_9PEZI | 26.85 | 108 | 66 | 4 | 68 | 170 | 50 | 149 | 7.00E-08 | 45.4 |
| VmHEP4 | A0A1V1T5J4_9FUNG | 26.57 | 143 | 90 | 6 | 34 | 171 | 6 | 138 | 2.00E-07 | 43.9 |
| VmHEP4 | A0A0G2HFC0_9PEZI | 25.23 | 111 | 74 | 3 | 62 | 171 | 71 | 173 | 1.00E-06 | 42 |
| VmHEP4 | W3WPK6_PESFW | 29.07 | 86 | 56 | 2 | 57 | 137 | 78 | 163 | 2.00E-06 | 41.6 |
| VmHEP4 | A0A1V6SXB7_9EURO | 26.28 | 137 | 80 | 7 | 50 | 179 | 6 | 128 | 5.00E-06 | 39.7 |
| VmHEP4 | A0A1Y2DR20_9PEZI | 23.64 | 110 | 74 | 3 | 62 | 170 | 75 | 175 | 6.00E-06 | 40 |
| VmHEP4 | A0A1R3RHQ9_ASPC5 | 25.9 | 139 | 80 | 5 | 26 | 152 | 1353 | 1480 | 1.00E-05 | 40.4 |
| VmHEP3 | A0A1C1WX61_9PEZI | 38.91 | 221 | 123 | 5 | 1 | 210 | 1 | 220 | 3.00E-43 | 142 |
| VmHEP3 | A0A0G2HFC0_9PEZI | 25.38 | 130 | 92 | 4 | 34 | 161 | 55 | 181 | 4.00E-08 | 46.6 |
| VmHEP3 | A0A1C1WQP7_9PEZI | 22.48 | 129 | 97 | 2 | 34 | 161 | 56 | 182 | 4.00E-08 | 46.6 |
| VmHEP3 | R0KMA8_SETT2 | 27.68 | 112 | 69 | 5 | 50 | 161 | 36 | 135 | 1.00E-06 | 41.2 |
| VmHEP3 | A0A179H6U1_9HYPO | 24 | 100 | 72 | 4 | 68 | 165 | 65 | 162 | 2.00E-06 | 41.6 |
| VmHEP3 | G9NWZ1_HYPAI | 27.18 | 103 | 71 | 4 | 50 | 152 | 41 | 139 | 3.00E-06 | 40.8 |
| VmHEP3 | A0A0W7W133_9HYPO | 27.18 | 103 | 71 | 4 | 50 | 152 | 41 | 139 | 3.00E-06 | 40.4 |
| VmHEP3 | A0A1Y2DR20_9PEZI | 25.37 | 134 | 89 | 5 | 27 | 153 | 47 | 176 | 5.00E-06 | 40.4 |
| VmHEP3 | T0LDB1_COLGC | 29.35 | 92 | 57 | 4 | 65 | 152 | 110 | 197 | 8.00E-06 | 40 |
| VmHEP3 | Q7RYC5_NEUCR | 27.19 | 114 | 74 | 4 | 48 | 158 | 95 | 202 | 9.00E-06 | 40 |
| VmHEP3 | G4UK32_NEUT9 | 27.19 | 114 | 74 | 4 | 48 | 158 | 95 | 202 | 9.00E-06 | 40 |

Table S2. Blast table shows the similarity between *V. mali* hce2s and their hce2 homologs, non-significant hits of hce2 homologs are filtered out. The table was output of BLASTP (protein-protein BLAST) with evalue=1e^-5^ in table format, *V. mali* Hce2s and all full length sequences (453 proteins) constructing Hce2 HMM model were used to make BLAST database. Query: Names of *V. mali* Hce2s; Subject: Names of *V.mali* Hce2s or Uniprot ID of Hce2 homologs; Q. Start and Q. Start: alignment start and end of query sequence; S. Start and S. End: alignment start and end of subject sequence.
