# Supplementary material for: Hce2 domain‐containing effectors contribute to the full virulence of Valsa mali in a redundant manner
Source: Mol Plant Pathol. 2019 Mar 26;20(6):843–56. doi: 10.1111/mpp.12796 (PMC6637899; doi:10.1111/mpp.12796)
Supplement: Supplementary file 9 — Table S3 Primers used in pSUC2 plasmid constructs in this study. [file MPP-20-843-s009.docx]

| Gene Number | Primer 5’-3’ F | Primer 5’-3’ R |
| --- | --- | --- |
| VM1G_00980 | CGGAATTCATGGCACGATTCACTATCGTC | CCGCTCGAGGCCGACGGCGAGGCTGG |
| VM1G_05547 | CGGAATTCATGAATCTCTTCGCCATCGTT | CCGCTCGAGGGCGATGACAAGGCAGGCCAGATTG |
| VM1G_07403 | CGGAATTCATGAAGTTTGCGACCATTCTCT | CCGCTCGAGAGCAAGAGCGGCCGAGGC |
| VM1G_09395 | CGGAATTCATGTCTTCCACCGTTCGAGC | CCGCTCGAGGGCGAGACAGACGGTCACC |
| VM1G_09394 | CGGAATTCATGCGTCTCACCACCTACG | CCGCTCGAGGGCGAAGCTGGTGCGTG |

**Table S3** Primers used in pSUC2 plasmid constructs in this study.
