# Supplementary material for: Hce2 domain‐containing effectors contribute to the full virulence of Valsa mali in a redundant manner
Source: Mol Plant Pathol. 2019 Mar 26;20(6):843–56. doi: 10.1111/mpp.12796 (PMC6637899; doi:10.1111/mpp.12796)
Supplement: Supplementary file 10 — Table S4 Primers for quantitative Reverse Transcription‐Polymerase Chain Reaction (qRT‐PCR) in this study. [file MPP-20-843-s010.docx]

| Gene | Primer for QRT-PCR/F | Primer for QRT-PCR/R |
| --- | --- | --- |
| G6PDH (*Valsa mali*) | TCAGAACAAGTTCGAGGGCGACAA | TGAGGGCAATAGAGGGCTTGTTCA |
| Vm1G_00980 | AGCCCATCTTCCGCAGCAT | CCGTTGTCGTCCGCCTTGA |
| Vm1G_05547 | TACTGCCCTAGCCAATTTCCA | GACCACCAGAGTCACCCTTCA |
| Vm1G_09394 | CCCCACCACCGTCAAGAAC | CAAGGTTGAAATGCGAGGC |
| Vm1G_09395 | AGTGGCGACATCGTGGTAG | AAGATCATCAACGGAAGTGAAA |
| Vm1G_07403 | CTCTTCCACGCCCTGACCT | GCCTTCACGGACCCAAACC |

**Table S4** Primers for qRT-PCR in this study.
