# Supplementary material for: Hce2 domain‐containing effectors contribute to the full virulence of Valsa mali in a redundant manner
Source: Mol Plant Pathol. 2019 Mar 26;20(6):843–56. doi: 10.1111/mpp.12796 (PMC6637899; doi:10.1111/mpp.12796)
Supplement: Supplementary file 11 — Table S5 Primers for gene deletion and Polymerase Chain Reaction (PCR) analysis in this study. [file MPP-20-843-s011.docx]

| Gene | Primer (5’-3’) |
| --- | --- |
| VmHEP1-1F | TAGCCAGCAGCCCTACCAG |
| VmHEP1-2R | GGCTTGGCTGGAGCTAGTGGAGGTCAATTCGCCCACTCACTTCAGC |
| VmHEP1-3F | AACCCGCGGTCGGCATCTACTCTATTCGGAAGCCATGATTAAACGG |
| VmHEP1-4R | ACTTGGGTCGCATTAGAGG |
| VmHEP1-5F | ATGCGTCTCACCACCTACGC |
| VmHEP1-6R | CTAGTTGGAACTACCAGAGTGACGT |
| VmHEP1-7F | GTGGCATGTGTGCATATTCT |
| VmHEP1-8R | AATAAAGGGAGGAAGGGCG |
| VmHEP1-9F | CAATACGAGGTCGCCAACAT |
| VmHEP1-10R | TACGAAGGGCACACTTTTATAA |
| VmHEP2-1F | GGGAGCGTTGCCTTGTTGG |
| VmHEP2-2R | GGCTTGGCTGGAGCTAGTGGAGGTCAAGTGGCTTTCTGGATGTTCCTTGA |
| VmHEP2-3F | AACCCGCGGTCGGCATCTACTCTATTCGGAATGGGTAACTTTGATGG |
| VmHEP2-4R | CAAGGGTTGTGGGTATGACT |
| VmHEP2-5F | ATGTCTTCCACCGTTCGAGC |
| VmHEP2-6R | CTACGGGAAATTGGCATCATC |
| VmHEP2-7F | TGGGGATGGAGATATCAAG |
| VmHEP2-8R | ATCGCGCATATGAAATCACG |
| VmHEP2-9F | GAACTGCCCGCTGTTCTGGA |
| VmHEP2-10R | TGGGATTTGATTTGGAGATC |
| VmHEP3-1F | AGATTAGCATTTGCGATTGAC |
| VmHEP3-2R | GGCTTGGCTGGAGCTAGTGGAGGTCAATGATGTTTGATTGCGACCT |
| VmHEP3-3F | AACCCGCGGTCGGCATCTACTCTATTCATGGGTTGATGTCGGTTAGAG |
| VmHEP3-4R | ACTTCCACAGCGGGTTCTC |
| VmHEP3-5F | ATGAATCTCTTCGCCATCGTT |
| VmHEP3-6R | CTAGACAGATGTCGTTGAGGACG |
| VmHEP3-7F | GGTGACTACCTAGGTATCATA |
| VmHEP3-8R | ATCGCGCATATGAAATC |
| VmHEP3-9F | TGCTGATCCCCATGTGTATC |
| VmHEP3-10R | CAGCCATCAATGTGATTCT |
| VmHEP4-1F | TATGGAGGACGATCAGGGTG |
| VmHEP4-2R | GGCTTGGCTGGAGCTAGTGGAGGTCAAGTCCGCAATGAGGCAAGAA |
| VmHEP4-3F | AACCCGCGGTCGGCATCTACTCTATTCAAGATATGAGTGCAATCGTGGAT |
| VmHEP4-4R | TCGTCCCTTGCCCAAATAG |
| VmHEP4-5F | ATGAAGTTTGCGACCATTCTCT |
| VmHEP4-6R | CTAAGGTCCACCAGATTCTTGTACA |
| VmHEP4-7F | TGACAACACCATCAACAACAGCG |
| VmHEP4-8R | GTCTGGCTAAGATCGGCCGCAG |
| VmHEP4-9F | TGCTGATCCCCATGTGTATCAC |
| VmHEP4-10R | GCGATGTGGCTCTGGTCGGTC |
| VmHEP5-1F | TGAGTTGAATAACCGCCTGCGTGAG |
| VmHEP5-2R | GGCTTGGCTGGAGCTAGTGGAGGTCAAGGATATTGTGAAGTGAATTACCG |
| VmHEP5-3F | AACCCGCGGTCGGCATCTACTCTATTCATGGATAGGTGGATATGTAGTATGG |
| VmHEP5-4R | CAGGTGGTCTTGGTTGGTG |
| VmHEP5-5F | ATGGCACGATTCACTATCGTC |
| VmHEP5-6R | TCAGTTCTCATAGTAGATCTTCCAC |
| VmHEP5-7F | CTCCGTCAAGTGATGGCATA |
| VmHEP5-8R | AGAGCCTGCGCGACGGACG |
| VmHEP5-9F | TGCTGATCCCCATGTGTATCA |
| VmHEP5-10R | CGGCTCTGAGCATCCATT |
| Hphcheck-F | CGCCCTTCCTCCCTTTATT |
| Hphcheck-R | GGTCGGCATCTACTCTATTCCTTT |
| Neocheck-F | CAGCCCGATTTCCATTCCT |
| Neocheck-R | CGGCGATACCGTAAAGCAC |
| Hph-F | GGCTTGGCTGGAGCTAGTG |
| Hph-R | AACCCGCGGTCGGCATCT |
| Neo-F | GAGGTTGCGATTTCTCTGC |
| Neo-R | GCCAGCAGTAGACACTTGGAA |

**Table S5** Primers for gene deletion and PCR analysis in this study.
